# Genetic and phenotypic analyses reveal major quantitative loci associated to fruit size and shape traits in a non-flat peach collection (*P. persica* L. Batsch)

**DOI:** 10.1038/s41438-021-00661-5

**Published:** 2021-11-01

**Authors:** Marco Cirilli, Irina Baccichet, Remo Chiozzotto, Cristian Silvestri, Laura Rossini, Daniele Bassi

**Affiliations:** 1 Università degli Studi di Milano - DiSAA, Milano, Italy; 2 Università degli Studi della Tuscia - DAFNE, Viterbo, Italy

**Keywords:** Plant breeding, Plant genetics

## Abstract

Fruit size and shape are critical agronomical and pomological attributes and prime targets in peach breeding programs. Apart from the flat peach type, a Mendelian trait well-characterized at the genetic level, ample diversity of fruit size and shapes is present across peach germplasms. Nevertheless, knowledge of the underlying genomic loci remains limited. In this work, fruit size and shape were assessed in a collection of non-flat peach accessions and selections, under controlled fruit load conditions. The architecture of these traits was then dissected by combining association and linkage mapping, revealing a major locus on the proximal end of chromosome 6 (*qSHL/Fs6.1*) explaining a large proportion of phenotypic variability for longitudinal shape and also affecting fruit size. A second major locus for fruit longitudinal shape (*qSHL5.1*), probably also affecting fruit size, was found co-localizing at locus G, suggesting pleiotropic effects of peach/nectarine traits. An additional QTL for fruit longitudinal shape (*qSHL6.2*) was identified in the distal end of chromosome 6 in a cross with an ornamental double-flower peach and co-localized with the *Di2* locus, controlling flower morphology. Besides assisting breeding activities, knowledge of loci controlling fruit size and shape paves the way for more in-depth studies aimed at the identification of underlying genetic variant(s).

## Introduction

Fruit external attributes play a crucial role in the definition of quality market standards. Size, color, shape, and absence of defects directly affect consumer’s preferences and acceptance. Not surprisingly, these traits continue to be the major breeding targets in horticultural crops^1^. Peach (*Prunus persica* L. Batsch) is the most cultivated species of the *Prunus* genus in temperate regions. Remarkable breeding efforts during the last century have led to a dramatic improvement of fruit appearance and the release of commercial cultivars with intense skin coloration, large size, and regular shapes^2^.

Basic descriptors such as height, length, width, and aspect ratios (i.e., length-to-width, height-to-length, or height-to-width), are major determinants of peach fruit shape. The optical impression of roundness by the human eye is associated with a height-to-width ratio of about 1.05, subtending to slightly oblate-shaped fruits^3^. Other shape characters include height and width of stalk cavity, tip-end presence and shape, suture prominence, and fruit symmetry^4^. In particular, suture deformation can modify the length-to-width ratio (also known as suture deformation index), causing a deviation from the spherical shape of the fruit^5^. Varietal improvement has privileged the selection of roundish (globose) fruits in almost all commercial peach and nectarine cultivars. The flat fruit type, known as ‘Pantao’ in China and ‘saucer’ or ‘donut’ peach in western countries, has been a notable exception, gaining a breeding interest starting from 1980 (with the release of the ‘Stark Saturn’ variety) and remaining a target trait in some breeding programs^6^. While peach cultivars are broadly classified into flat or non-flat (or round) types, a huge variability of fruit shapes have been reported across non-flat peach accessions, widely varying from oblate to ovate/elongated. Based on longitudinal (parallel or perpendicular to the suture line) and transversal sections, Blake & Edgerton^7^ classified peach standards into 12 classes for the longitudinal section and 9 for the transversal one, respectively. While being part of the routine breeding evaluation, fruit shape attributes have rarely been evaluated through objective quantitative measurements (e.g., using a caliper or image-based tools) and roughly categorized through visual assessment, particularly focusing on the overall shape and/or the scoring of tip and suture prominence^8^.

Genetic analysis of peach fruit shape mainly focused on the Mendelian flat trait controlled by the dominant *S* locus initially described by Lesley^9^ and fine-mapped to the distal part of chromosome 6^10,11,12,13^. Apart from this locus, knowledge of other genetic factors contributing to shape variation in non-flat-type fruits is still limited. A moderate correlation with fruit size and an intermediate heritability estimate have been reported for the ratio between height and average suture and cheek diameter^8^. A genotypic contribution to fruit shape is supported by the distribution of round and ovate shapes in some breeding progenies^5^. Blake^14^ suggested a dominance of ovate over round shape, in contrast to Smykov^15^, reporting an almost fully inherited roundish over ovate shapes from visual analysis of several segregating progenies. A more quantitative inheritance has been proposed based on the wide range of variability obtained by crossing round and ovate cultivars, albeit with a slight bias towards round^3^. Genetic analysis of fruit shape may be complicated by the influence of environmental conditions—in particular pre- and post-blooming temperatures seem to strongly influence pistil tip and suture prominence^16^. For example, high-chill peach cultivars grown in tropical climate often show elliptic (i.e., heart-shaped) fruit with accentuated tip and/or suture bulge^17^, suggesting a role of inadequate chilling or prolonged endo-dormancy on flower bud and early ovary development^18^. Analysis of some progenies from low-chill breeding programs suggests low heritability of these traits and strong genotype-by-environment interaction^19^. Studies on the effect of tree cultivation management on fruit shape are scarce, although excessive fruit thinning seems to increase the incidence of fruit deformation, at least in some cultivars^20^.

Fruit size is another important external quality attribute but also a component of yield and, thus, a fundamental characteristic for developing novel cultivars. Peach fruit size is generally estimated measuring equatorial diameter, often highly correlated with fruit mass (i.e., fresh weight) within a genotype^8,21^. However, differences in endocarp size (i.e., flesh to pit ratio)^22^ or fruit shape (e.g., in flat fruits) among genotypes could bias the correlation between fruit size and mass, making them de facto well-distinct traits. Fruit size in stone fruits is a complex trait affected by genotype, environment, and their interactions^8,19^. Also, orchard management, including rootstock, fertilization, irrigation, and/or pruning has significant effects on this trait^23^. At physiological level, final drupe size is a function of mesocarp cell number, cell size, and intercellular space^24,25^. Fruit growing potential is primarily determined by cell number (although cell size can differ among varieties), which in turn depends on genetic factors (possibly related to the number of ovary cells at bloom)^26–28^ and source-sink limitations occurring during specific growth phases (driven by the competition for assimilates among fruits)^29–31^. Assimilates supply is controlled through the adjustment of fruit load by thinning (i.e., removal of flowers or fruitlets), a cultivation practice with a tremendous effect on fruit size, particularly on genotypes with high growth potential^32^. At genetic level, a wide range of heritability estimates have been reported for peach size (suture and cheek diameter), varying from lower than 0.30 to higher than 0.90 depending on the study and occurrence of transgressive segregation^8,16,19,21,33,34^. Apart from population-specific genetic factors, such differences in heritability estimates may at least in part be due to differences in environmental conditions and tree management practices (i.e., fruit load)^35^. The knowledge of the genetic bases underlying fruit size variation would bring significant benefits to the selection process, including markers or genomic assisted approaches. QTL mapping studies for fruit size have been performed in peach as well as other stone fruits, most of them using bi-parental progenies or pedigree-based approaches. In peach, QTLs for fruit size have been reported in inter- and intra-specific progenies on chromosomes 1, 2, 3, 4, 5, 6, and 7, although their presence, position, and/or explained phenotypic variance widely differed depending on the genetic background used for dissection^21,22,33,34^. In other *Prunus* species, such as sweet cherry, major overlapping QTLs for drupe size were repeatedly found on chromosomes 2 and 6, with additional loci reported on 1 and 5^25,36,37^.

Objective of this study was to dissect the genetic architecture of fruit size and shape traits in a collection of 172 non-flat accessions and selections representative of occidental peach cultivation and breeding. To this end, the collection was phenotyped using objective measures and indices during two consecutive seasons under controlled crop load conditions. Association mapping revealed several loci associated with fruit size and shape, including a major locus on the proximal end of chromosome 6 (*qSHL/Fs6.1*) explaining most of the phenotypic variability for longitudinal shape and also affecting fruit size. The effects of this QTL on fruit longitudinal shape were confirmed in two different segregating progenies, while those on fruit size seem more dependent on the genetic background. An additional QTL for longitudinal shape (*qSHL5.1*) was identified on chr 5 co-localizing with the *G* locus, suggesting a putative pleiotropic effect of the peach/nectarine trait. Finally, a QTL for longitudinal shape (*qSHL6.2*) was identified in a cross-progeny derived from an ornamental double-flower peach and co-localized with the *Di2* locus controlling flower morphology.

## Results

### Characterization of fruit size and shape in a peach breeding collection

**Fig. 1:**
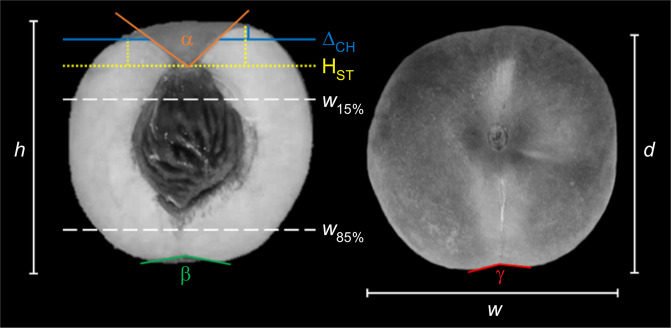
Graphical description of fruit morphological parameters measured in the collection panel. Graphical description of fruit morphological parameters measured in the collection panel. Fruit height (h), width (w) and depth (d), in millimeters (mm); proximal and distal width (*w*_*15%*_, *w*_85%,_ respectively); height of stalkcavity (*H*) and proximal indentation (*Δ*_*CH*_), as relative height difference between cheek points, in millimeters(mm); suture indentation (*γ*), width of stalk cavity (*α*) and indentation angle (*β*), in angle degree (°)

Quantitative data for fruit size and shape were estimated by using standard indices and others relevant for peach drupe (schematically shown in Fig. 1). The main parameters were maximum fruit height (*h*, the distance between apex and maximum cheek point) width (*w*, the distance between the two cheeks of the fruit), and depth (*d*, the distance between suture line and its opposite side). Fruit size (*Fs*) was determined by averaging maximum *w* and *d*. The *w*/*h* (*longitudinal shape index, SH*_*L*_) and *w*/*d* (*transversal shape index*, *SH*_*T*_) ratios provide estimates of fruit shape in the longitudinal and transversal sections, respectively. For both indices, a value of 1.05 is associated with a visual perception of round fruits, while lower or higher values correspond to ovate and oblate shapes, respectively. In the longitudinal section, the ratio of proximal to distal width (*w*_15%_/*w*_85%_, *longitudinal shape triangle index, ST*_*L*_) allows differentiating the ovate from the elliptic (heart-shaped) form. The same could be also applied to the transversal section (*transversal shape triangularity index, ST*_*T*_). In the transversal section, the degree of suture indentation was also visually scored (γ). Proximal fruit end shape was evaluated by the indices: *width of stalk cavity* (*α*), estimated as the angle formed by the top cheeks points and the site of stalk attachment; *height of stalk cavity* (*H*_*ST*_) as the vertical distance between the stalk attachment point and the tangent line to the maximum cheeks points; *proximal indentation* (*Δ*_*CH*_) as the relative height difference between cheek points (*Δ*_*CH*_ is also a proxy measure of cheeks asymmetry). In the distal fruit end, tip length and (in case of tip absence) the *indentation angle* formed by the distal cheek points and fruit apex (*β*), were also evaluated.

**Fig. 2:**
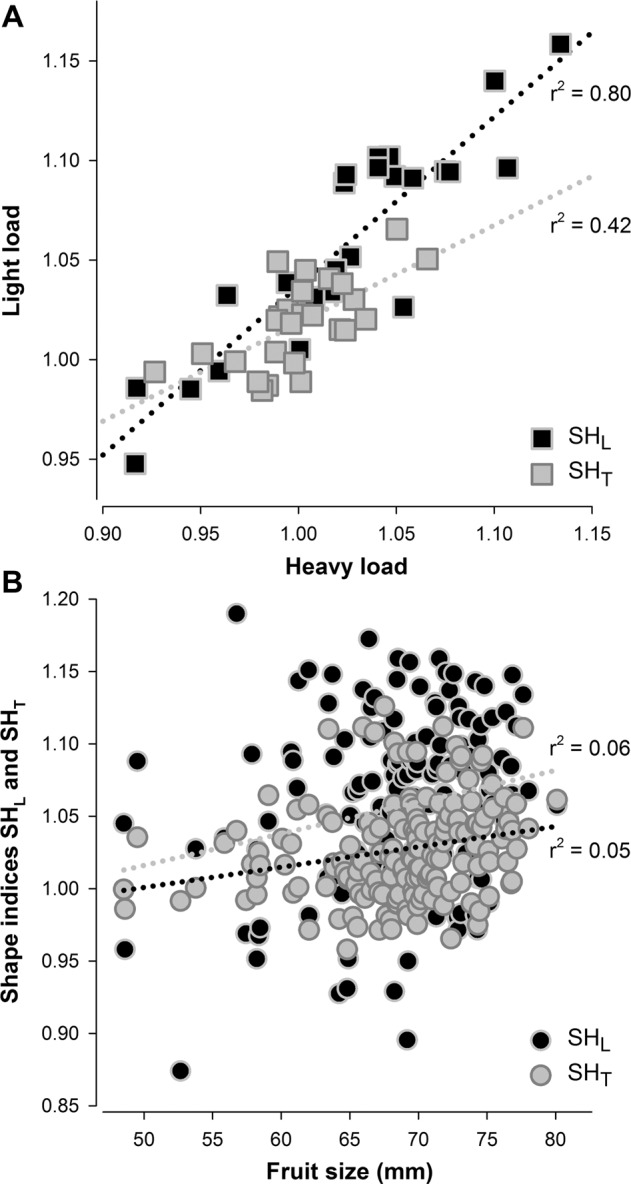
Relationships of crop load and fruit size with fruit shape parameters. **A** Effect of crop load (CL) treatment on fruit longitudinal (*SH*_*L*_) and transversal (*SH*_*T*_) shape. Light, 1 fruit per cm^2^ of TCSA (Trunk Cross Sectional Area), and heavy, 3 fruit per cm^2^ of TCSA treatments were compared using Pearson correlation in a subset of 26 accessions. **B** Relationship between fruit size (measured at CL = 1) and fruit longitudinal (*SH*_*L*_) and transversal (*SH*_*T*_) shape in the whole collection panel

As the first line of investigation, considering the well-known effect of crop load (CL) on fruit size, two thinning treatments—i.e., light and heavy, based on fruits number per cm^2^ of Trunk Cross-Sectional Area in the range 0.7–1 (light) and 2–2.5 (heavy)—were applied in a subset of 26 accessions representative of the range of size and shape variability. As expected, the reduction of fruit load significantly increases fruit size (*Fs*), with a magnitude dependent on the maximum growth potential of each accession (from 5 to 14 mm on average). Interestingly, fruit load also affects fruit shape, increasing *SH*_*L*_ (*r*-squared of 0.80, *p* < 0.01) independently from the year or genotype, while *SH*_*T*_ was less affected (*r*-squared of 0.42, *p* < 0.10) (Fig. 2). Therefore, to account for size effects on fruit shape, all accessions were subjected to the same light thinning treatment, in order to limit within-tree competition and ensure fruit comparisons under conditions of optimum growth potential.

**Fig. 3:**
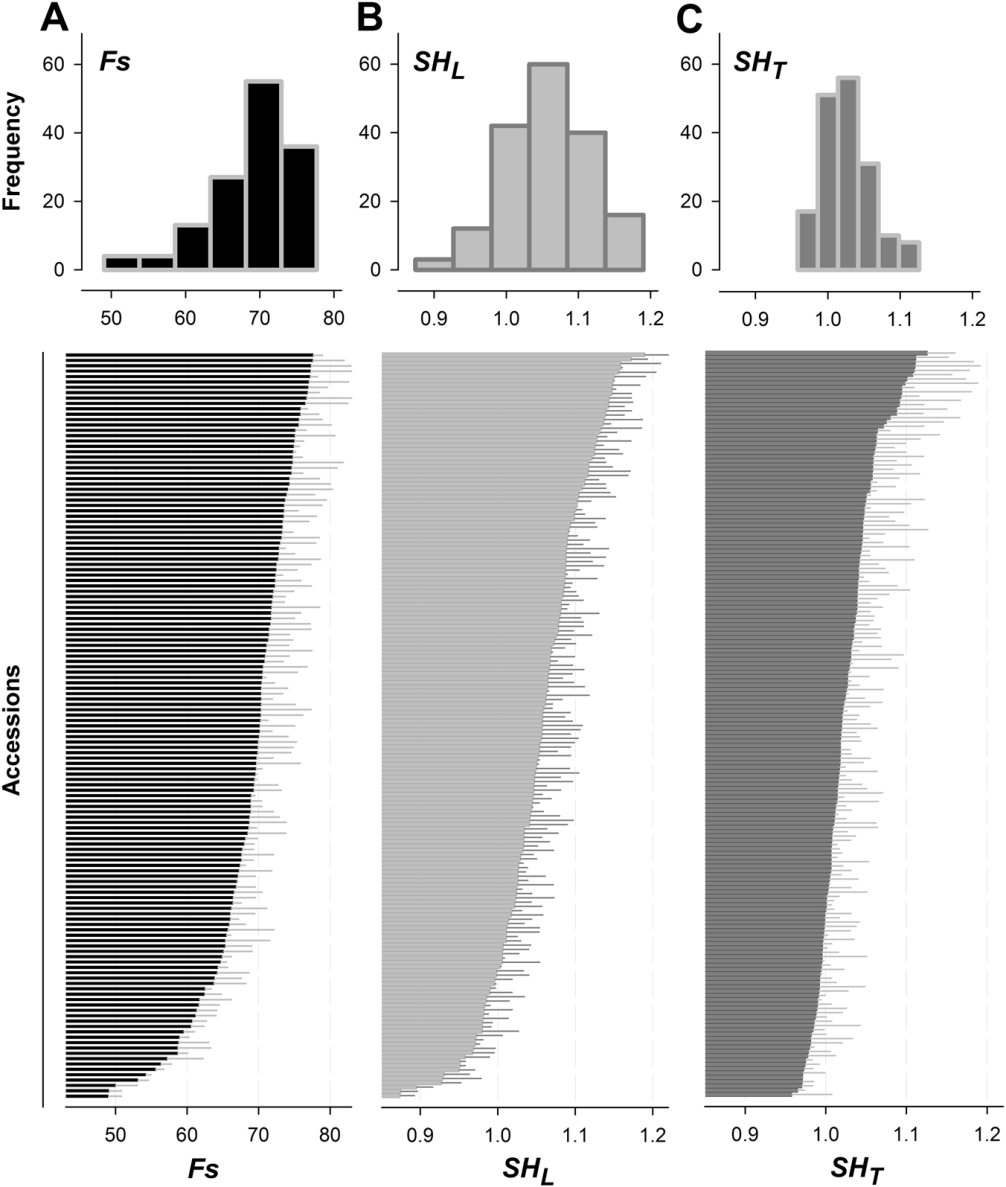
Distributions of fruit size and shape parameters. Frequency distribution, mean and standard deviation of **A** fruit size (*Fs*), **B** longitudinal shape (*SH*_*L*_) and **C** transversal shape (*SH*_*T*_) in the collection panel. Data are the average of 2 years of evaluation

In the whole panel, no evident correlation between *Fs* and both *SH*_*L*_ and *SH*_*T*_ were observed under these load conditions (Fig. 2). Overall, drupes showed a remarkable diversity for size (*Fs*), ranging from 49.0 mm to a maximum of 80.6 mm (Fig. 3A), highly correlated in the 2-years of evaluation (*r*-squared 0.82) (Supplementary Fig. 1).

**Fig. 4:**
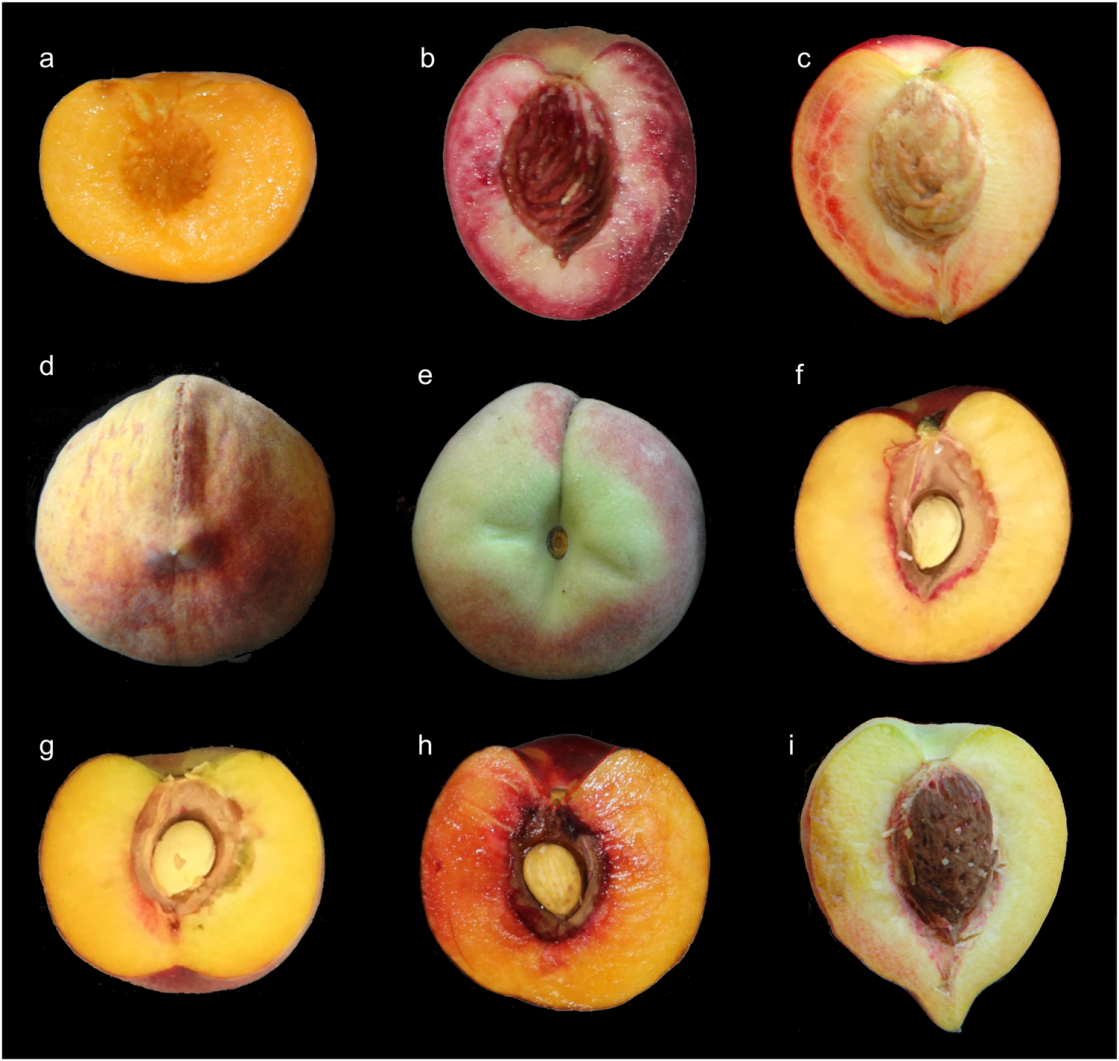
Overview of peach accessions showing extreme range of phenotypic variation for morphological traits. Longitudinal shape (*SH*_*L*_) oblate in ‘Oro A’ (**a**) and ovate in ‘Carota’ (**b**); longitudinal shape triangle index (*ST*_*L*_), elliptic (heart-shaped) in ‘Pacific Star’ (**c**); transversal shape triangularity index (*ST*_*T*_), triangular-shaped fruits in ‘Pillar’ (**d**); evident deep suture indentation (*γ*) in ‘Buco Incavato’ (**e**); cheeks asymmetry (*Δ*_*CH*_) in ‘Early Top' (**f**); width of stalk cavity (*α*), wide in ‘KV930386’ (**g**) and narrow in ‘BO05020077’ (**h**); pronounced tip in ‘Pesca a Cuore Capuozzi’ (**i**)

*SH*_*L*_ widely range from 0.86 to 1.19 (across-years averages), although most of the accessions were characterized by nearly round-shaped fruits (i.e., in the range 1.00–1.05) (Fig. 3B). The upper and lower limits of this interval were measured in the accessions ‘Oro A’ and ‘Carota’, characterized by an oblate and ovate shape, respectively (Fig. 4A, B). *SH*_*L*_ values were highly correlated across the 2-years of observations (*r*-squared = 0.73) (Supplementary Fig. 1). Notably, none of the 53 nectarine accessions in our panel showed *SH*_*L*_ values higher than 1.09, except for the oblate L1 nectarine chimera ‘Angelo Marzocchella’ (a sport mutation of ‘Vesuvio’), with an average of 1.17.

*SH*_*T*_ showed a lower range of variation (0.96 and 1.13, across-years averages) compared to *SH*_*L*_ (Fig. 3C). The upper and lower limits of this range were observed in ‘Tasty Free’ (slightly elliptic) and ‘Xia Hui’ (slightly oblate). However, this index showed a moderate correlation between the 2-years (*r*-squared = 0.45, *p* < 0.10) (Supplementary Fig. 1).

*ST*_*L*_ continuously varied between 1.00 and 1.27 (being value higher than 1.20 visually perceived as elliptic), although it was affected by high within-accession variability (pooled standard deviation = 0.082) and low correlation between-years (*r*-squared = 0.34, *p* < 0.10) (Supplementary Figs. 1 and 2). The maximum value of this index was observed in accession ‘Pacific Star’ (Fig. 4C) while obovate (pear-like) shapes (i.e., *ST*_*L*_ values lower than 1) reported in old classification systems for peach was not present in the collection. Regarding *ST*_*T*_ in the transversal section, fruits showed no appreciable variability, except for the out-ranking accessions ‘Pillar’ and ‘Groc d’Alto’, having a high incidence of triangular-shaped fruits (caused by tissues swelling at the suture level) (Fig. 4D). Also, suture indentation (*γ*) did not show appreciable variability, being flat or slightly shallow in almost all accessions, excluding ‘Buco Incavato’, with an evident and deep indentation (Fig. 4E).

The other parameters showed moderate to low correlation among years, ranging from 0.57 for *α* to 0.37 for *H*_*ST*_ (Supplementary Fig. 1). *Δ*_*CH*_ index was characterized by a minor range of variation, assuming values from less than 1 mm in ‘Early Top’ to 7.9 mm in ‘BO99003001’—the former showing cheeks asymmetry (Fig. 4F and Supplementary Fig. 1); α ranged between the wide angle of 127.1° in ‘KV930386’ to narrow angle of 67.2° in the selection ‘BO05020077’ (Fig. 4G, H and Supplementary Fig. 1), while *H*_*ST*_ from 5.11 to 13.8, respectively, in ‘Borgia’ and in ‘Royal Majestic’. Regarding distal fruit end shape, the incidence of protruding tip was negligible, as in most of the accessions it was either absent or only slightly sketched (<2–3 mm), except for ‘Pesca a Cuore Capuozzi’, consistently characterized by a stylar tip length over 10 mm (Fig. 4I). Also, in fruit without tip, the distal indentation angle (*β*) showed low variability among accessions, being flat or slightly depressed (data not shown).

The correlation among the various indices was also evaluated by Spearman’s test; however, excluding the negative correlation between *α* and *H*_*ST*_ (*r*-squared −0.61), all other parameters were not significantly related.

### Population structure and PCA of fruit size and shape

As inferred by the Admixture program, the predictive accuracy of population structure in the panel was maximized for two a priori clusters (K) (Supplementary Fig. 3). For a membership coefficient higher than 0.7, almost half of the genotypes were assigned to the Occidental breeding cluster (group 1), which includes commercial varieties (including most of the nectarines) and derived selections^35^. The second cluster (group 2) included various genetic materials, such as Occidental traditional accessions, early USA breeding materials, and a few individuals with Oriental origins. The remaining were admixed genotypes. In Principal component analysis (PCA) of size and shape, the 2 first principal components were found to be relevant following the scree plot method, accounting for 51.8% of the total variation. The first and second PC account for 31.4% and 20.4%, respectively (Supplementary Fig. 4). The first component (*Dim1*) was correlated to *H*_*ST*_, *Fs*, and *α* (−0.58, −0.52, and 0.44, respectively) while the second (*Dim2*) to the main shape attributes *SH*_*L*_, *SH*_*T*_, and *ST*_*L*_ (−0.56, −0.65, and −0.38, respectively). The projections of the accessions on the PCA graph show no evident differentiation among clusters, maybe except for some genotypes of group 1 (accessions of Occidental breeding) characterized by negative values of Dim1, and of group 2 presenting high positive values for Dim2.

### GWAS analysis

As a proof-of-concept of the statistical power of the 18K SNP array for GWAS, we analyzed the already characterized non-flat/flat trait (S locus), by adding 11 flat genotypes to the panel of 172 non-flat accessions. Based on previously published mapping information, the best accuracy was achieved using the FarmCPU algorithm adjusted for population structure (Q matrix for *K* = 2), identifying SNP_IGA_683904 as the most associated marker at position 25,033,223 bp (Supplementary Fig. 5), consistent with previous report^38^: this marker is about 1.8 Mb from the most promising candidate variant, a 1.67 Mb chromosomal inversion^12^.

Next, GWAS was conducted in the panel of 172 non-flat accessions with different statistical models (MLMM, FarmCPU, and Blink) and using the average of yearly records and associations were detected for size and shape traits.

**Fig. 5:**
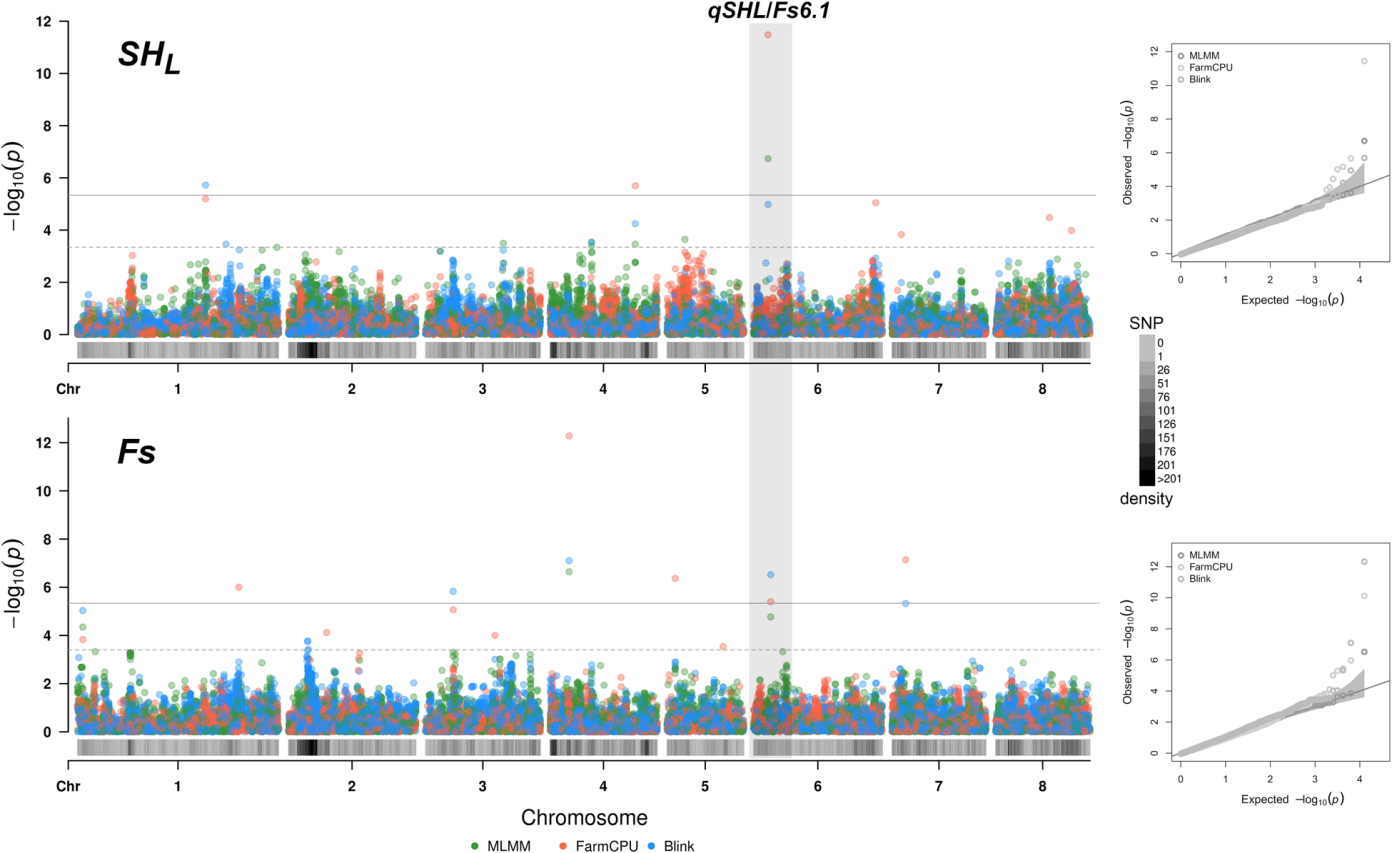
Association mapping for fruit longitudinal shape (*SH*_*L*_) and fruit size (*Fs*) traits. Manhattan and QQ plots of -log *p* values estimated from 2-year averaged data using Blink, FarmCPU, and MLMM models adjusted for population structure (*K* = 2). Horizontal lines indicate the Bonferroni-adjusted threshold (continuous line, 4.01e^−06^) and *permutation* test (dashed lines). The position of *qSHL/Fs6.1* locus is highlighted

For longitudinal shape index (*SH*_*L*_), signals at the proximal end of chromosome (chr) 6 were detected by all models and in both years, although the top SNPs were different: Peach_AO_0600284 for 2018 (3,021,600 bp, max *p*-value of 6.01e^−12^) and Peach_AO_0601598 for 2019 (3,440,645 bp, max *p*-value of 6.01e^−12^) (Table 1). Other signals were identified on chr 1, 3, 4, 6, and 8, depending on year or model. Considering the high correlation of *SH*_*L*_ between-years (0.77), average values were also used for the analysis, confirming the main locus at the beginning of chr 6 with top marker at SNP_IGA_609531 (3,499,037 bp, max *p*-value of 3.32e^−12^). Other loci above the Bonferroni threshold were detected on chr 1 and 4 (Table 1 and Fig. 5). Cumulative heritability explained by GWAS loci using average data was 0.59.

**Table 1:** Statistical information on associated SNPs from GWAS analysis for morphological traits

Trait	SNP id	Chr	Pos	MAF	*p*-value	Model
2018						
*SH*_*L*_	Peach_AO_0600284	6*	3,021,600	0.26	6.01e^−12^	B, F, M
	Peach_AO_0490782	4	20,290,630	0.27	1.59e^−08^	B
	Peach_AO_0614799	6	8,235,462	0.10	6.02e^−07^	B
	Peach_AO_0377861	3	18,517,150	0.05	1.14e^−06^	B, F
	Peach_AO_0094708	1	30,479,453	0.21	8.61e^−06^	B
*Fs*	Peach_AO_0424020	4	4,578,765	0.09	2.66e^−06^	B, F, M
	SNP_IGA_605104	6*	4,132,770	0.24	3.16e^−06^	B, F, M
	Peach_AO_0535904	5	2,028,835	0.17	4.29e^−06^	F
*SH*_*T*_	Peach_AO_0590213	5	16,735,967	0.26	4.10e^−04^	B
	SNP_IGA_825124	8	5,637,193	0.12	4.72e^−04^	B, F, M
α	SNP_IGA_7584	1	2,350,054	0.27	1.08e^−04^	M
	Peach_AO_0575699	5	11,039,355	0.19	5.99e^−04^	F
*Δ*_*CH*_	SNP_IGA_864911	8	14,668,631	0.40	5.68e^−04^	B, F, M
*H*_*st*_	Peach_AO_0836299	8	11,002,921	0.38	2.96e^−04^	B, F, M
	SNP_IGA_499833	4	21,722,367	0.38	3.01e^−04^	B, F, M
2019						
*SH*_*L*_	Peach_AO_0601598	6*	3,440,645	0.30	2.31e^−17^	B, F, M
	Peach_AO_0844595	8	12,910,222	0.14	2.10e^−06^	F
*Fs*	Peach_AO_0424020	4	4,578,765	0.09	1.76e^−11^	B, F, M
	SNP_IGA_605104	6*	4,132,770	0.24	5.21e^−07^	B, F, M
	SNP_IGA_757328	7	10,115,708	0.32	8.04e^−06^	B
	Peach_AO_0003399	1	1,310,268	0.09	1.03e^−06^	B, F, M
*SH*_*T*_	SNP_IGA_19514	1	6,872,054	0.15	1.78e^−04^	B, F, M
	SNP_IGA_817953	8	4,535,051	0.27	2.25e^−04^	B, F, M
* ST*_*L*_	Peach_AO_0401556	3	25,678,981	0.18	1.16e^−04^	M
	SNP_IGA_693205	6	27,589,956	0.22	9.27e^−04^	B, M, F
*α*	SNP_IGA_856552	8	5,637,193	0.15	1.96e^−04^	M
	Peach_AO_0583798	5	13,948,395	0.40	6.83e^−04^	M
*Δ*_*CH*_	SNP_IGA_864911	8	14,668,631	0.40	5.68e^−04^	B, F, M
Average						
*SH*_*L*_	SNP_IGA_609531	6*	3,499,037	0.29	3.32e^−12^	F, M, B,
	Peach_AO_0094708	1	30,479,453	0.21	1.89e^−06^	B, F, M
	Peach_AO_0490782	4	20,290,630	0.27	2.00e^−06^	F, B, M
*Fs*	Peach_AO_0424020	4	4,578,765	0.09	5.40e^−13^	B, F, M
	Peach_AO_0707424	7	3,229,951	0.37	7.22e^−08^	F, B
	SNP_IGA_605104	6*	4,132,770	0.24	3.01e^−07^	B, F, M
	Peach_AO_0535904	5	2,028,835	0.17	4.29e^−07^	F
	SNP_IGA_114495	1	38,398,691	0.26	9.93e^−07^	F
	SNP_IGA_309724	3	6,615,375	0.28	4.63e^−06^	B, F
	Peach_AO_0003399	1	1,310,268	0.09	9.27e^−06^	B, M

*Asterisks indicate the *qSHL/Fs6.1* QTL. GWAS algorithm abbreviations: *B*, Blink; *F*, FarmCPU; *M*, MLMM

For fruit size (*Fs*), stable loci were identified at the same marker positions by all models on the proximal ends of chr 4 and 6: Peach_AO_0424020, located at 4,578,765 (max *p*-value of 2.66e^−06^ and 1.76e^−11^, respectively, for 2018 and 2019) and SNP_IGA_605104, located at 4,132,770 bp (max *p*-value of 3.16e^−06^ and 5.21e^−07^, respectively) (Table 1). Other associations were identified on chr 1, 5, and 7, depending on the year or model. Also for *Fs* trait, the high between-years correlation (*r*-squared 0.70) allows testing average phenotypic values: other than the two major loci on chr 4 and 6, several significant signals were detected on chr 1 (SNP_IGA_114495, at 38,398,691 bp and Peach_AO_0003399 at 1,310,268 bp), 3, 5, and 7 (Table 1 and Fig. 5). Heritability explained by GWAS loci using average data was 0.60.

Regarding the other shape traits, only signals above the respective permutation thresholds were identified, mostly depending on the year (Table 1). Cumulative heritability was lower compared to *Fs* and *SH*_*L*_, ranging from 0.28 for *SH*_*T*_ to 0.15 for *Δ*_*CH*_. For transversal shape index (*SH*_*T*_), stable signals were identified by all models on chr 8, at markers SNP_IGA_825124 (5,637,193 bp) for 2018 and SNP_IGA_817953 (4,535,051 bp) for 2019 with a maximum *p*-value of 4.72e^−04^ and 2.25e^−04^, respectively, above the permutation threshold of 8.49e^−04^. For proximal indentation (*Δ*_*CH*_) a stable association in both years was detected by all models on chr 8 at SNP_IGA_864911, located at 14,668,631 (maximum *p*-value 5.68e^−04^) above the permutation threshold (3.84e^−03^). For the width of stalk cavity (*α*), associated loci were detected on chr 1 and 5 for 2018, and chr 5 and 8 for 2019. Finally, for the height of stalk cavity (*H*_*ST*_) and shape triangle index (*ST*_*L*_) signals were only identified for 2018 or 2019, respectively.

### Association of *qSHL/Fs6.1* with fruit size and shape in the accession panel and breeding progenies

**Fig. 6:**
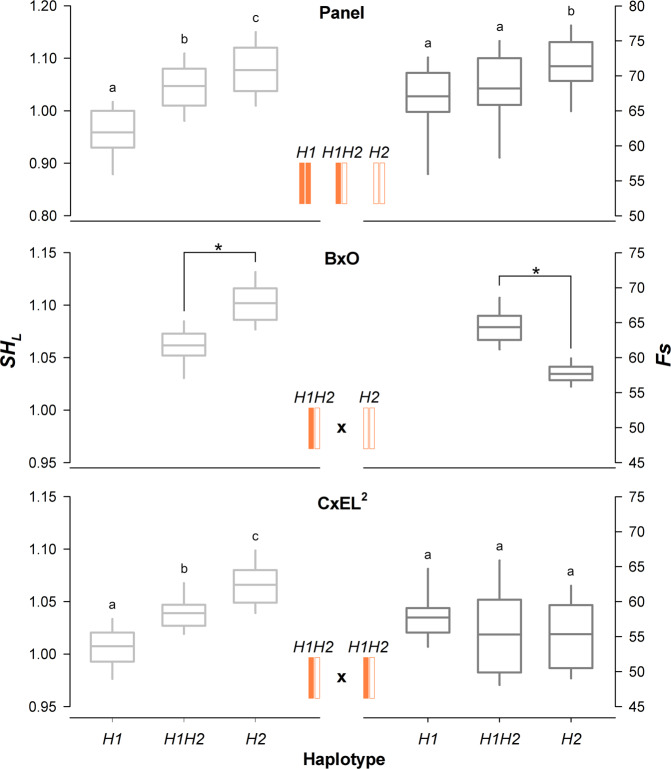
Boxplots of the associations between the three prevalent haplotypes (H1, H1H2, and H2) at *qSHL/Fs6.1* locus with fruit longitudinal shape (*SH*_*L*_) and size (*Fs*) in the collection panel, BxO and CxEL^2^ progenies. Letters and asterisks indicate significant differences between segregating classes (*p* < 0.01) as inferred by one-way ANOVA and Student&requo;s *t* test

Among the stable and highly significant genomic regions identified by GWAS, the proximal end of chr 6 was associated to both fruit size and longitudinal shape. In particular, the peak corresponding to marker SNP_IGA_605104 (4,132,770) for *Fs* trait is close to the signals identified for *SH*_*L*_ (SNP_IGA_609531, at 3,499,037 bp). Associated markers map to a local high LD region spanning about 1.2 Mb delimited by Peach_AO_0600618 and Peach_AO_0605368 (3,084,906–4,211,505 bp), named *qSHL*/*Fs6.1* (Supplementary Fig. 6). Among the different SNP haplotype blocks defining this LD region, only three are common to more than 87% of accessions in the panel: homozygous for H1 or H2 haplotypes or heterozygous (i.e., non-recombinant H1H2) (Supplementary File 1). Blocks H1, H1H2, and H2 were significantly associated with *SH*_*L*_, showing an ovate (0.96), round (1.05), and slightly oblate (1.08) shapes, respectively, and explaining 31.6% of the phenotypic variance in the panel. Association of these haplotypes with *Fs* trait was only significant for H2, being associated with an increase of drupe size (Fig. 6).

To further explore the involvement of *qSHL*/*Fs6.1* locus, two bi-parental progenies segregating for different SNP blocks were investigated (Supplementary File 1): an F_1_ progeny (BxO) from the cross ‘Bolero’ [B, round (*SH*_*L*_ = 1.01), large size (*Fs* = 74.2 mm)] × ‘Oro A’ [O, oblate (*SH*_*L*_ = 1.19), small size (*Fs* = 57.2 mm)]; an F_2_ progeny (CxEL^2^) derived from cross ‘Contender’ [C, round (*SH*_*L*_ = 1.05), medium size (*Fs* = 69.7 mm)] × ‘Elegant Lady’ [D, slightly oblate (*SH*_*L*_ = 1.09), large size (*Fs* = 75.0 mm)].

In BxO, *Fs* and *SH*_*L*_ traits were highly to moderately correlated in the two analyzed seasons (*r*-squared 0.78 and 0.60, respectively), with broad-sense heritability of 0.62 and 0.58, respectively. *SH*_*T*_ did not show appreciable variability (data not shown). Average *Fs* values showed an almost bimodal distribution, while *SH*_*L*_ was normal but negatively skewed. Interestingly, the two traits were correlated (*r*-squared −0.78), as fruit size increase along with the decrease of *SH*_*L*_ (i.e., in rounder fruits). Linkage analyses in the BxO map detected a major QTL at the proximal end of chromosome 6, peaking for both *Fs* and *SH*_*L*_ traits at SNP_IGA_608970 (located at 3,763,505 bp and co-localized within *qSHL*/*Fs6.1*), with maximum LOD of 28.2 and *K*-score of 41.12, respectively (Supplementary Fig. 7). This QTL explained 64.2% and 46.8% of phenotypic variance for *SH*_*L*_ and *Fs*, respectively (Fig. 6). No additional QTLs were detected in other chromosomes. BxO seedlings segregated 1:1 (chi-square = 0.83) for the H1H2 (B parent) and H2 (O parent) blocks at *qSHL*/*Fs6.1*, respectively, associated to round-oblate and oblate (averages of 1.05 and 1.11). Interestingly, the H1H2 is also associated with increased fruit size compared to H2 (averages of 65 and 57 mm, respectively).

CxEL^2^ seedlings segregated 1:2:1 (chi-square = 1.60) for the H1, H1H2, and H2 blocks, respectively; broad-sense heritability of *Fs* and *SH*_*L*_ was of 0.61 and 0.30, respectively. Even in this progeny, 1-year phenotyping revealed a significant association between *qSHL*/*Fs6.1* segregation and the range of *SH*_*L*_ value, varying from about 1.00–1.07 on average. In contrast, effects of this QTL on fruit size were not significant in this progeny (Fig. 6).

### Putative pleiotropic effects of skin hairiness and maturity date on peach size and shape

**Fig. 7:**
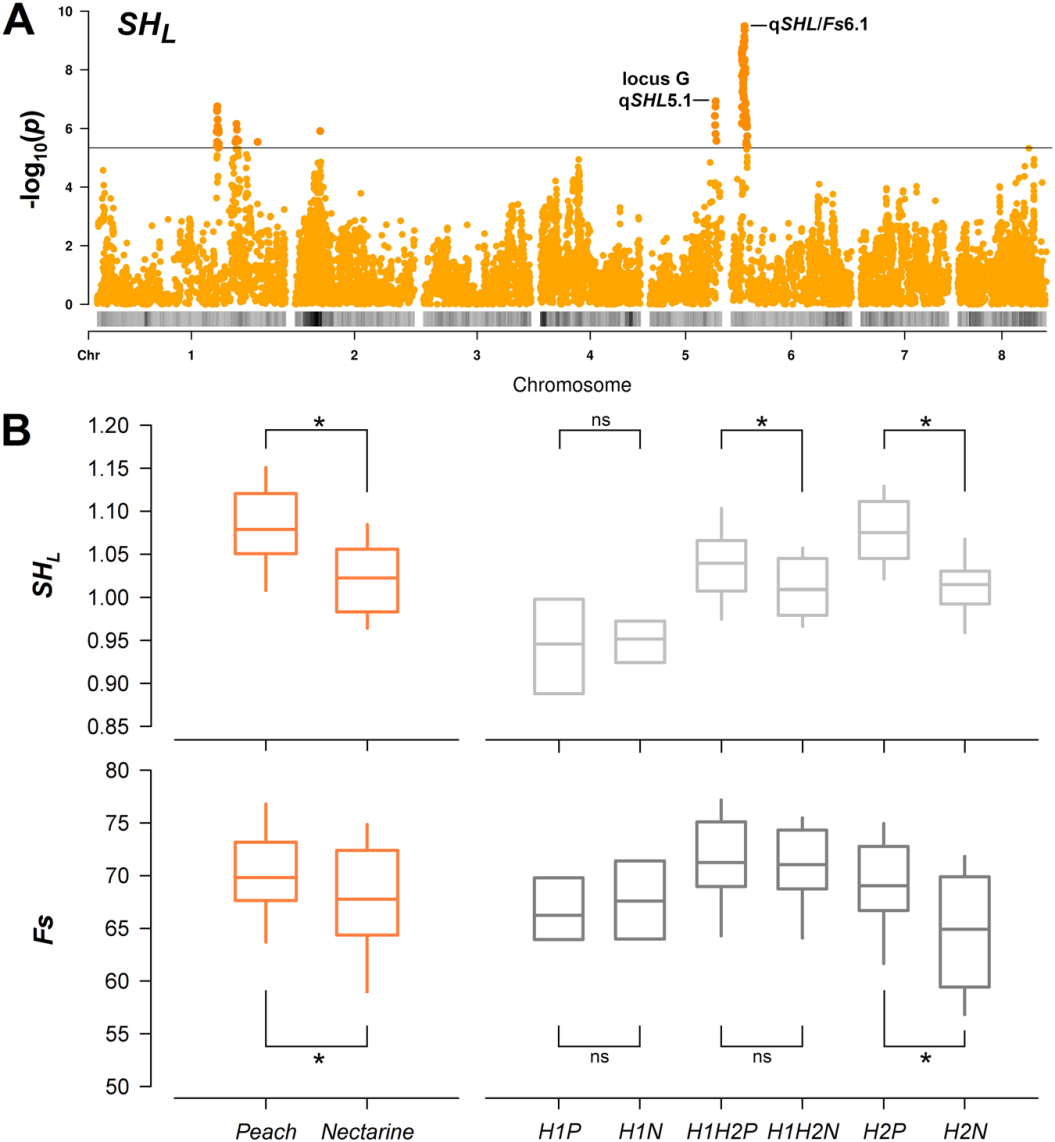
Association mapping of peach fruit size and shape. **A** Association mapping for fruit longitudinal shape (*SH*_*L*_) using naive GLM model. Horizontal lines indicate theBonferroni-adjusted threshold (continuous line, 4.01e^−06^). The position of *qSHL5.1/locus G* and *qSHL/Fs6.1* locus is highlighted. **B** Boxplots of the associations between the combination of the haplotypes at *qSHL/Fs6.1* locus (H1, H1H2, and H2) and peach/nectarine trait (*P* and *N*, respectively) with fruit longitudinal shape (*SH*_*L*_) and size (*Fs*) in the collection panel. Asterisks indicate significant differences between pairwise groups (*p* < 0.01), as inferred by Student’s *t* test (peach versus nectarine) and one-way ANOVA (haplotypes groups)

For *SH*_*L*_ trait, no signals were detected by GWAS close to the *G* locus controlling peach/nectarine trait, located at about 15.9 Mb on chr 5^39^. However, significant differences were found between peach and nectarine accessions groups, with average *SH*_*L*_ of 1.075 and 1.019, respectively (Fig. 7). Since most nectarine accessions were included in the Occidental breeding cluster, association analysis was performed without adjusting for population structure. A major QTL was found by naive GLM model at the distal end of chromosome 5 (*qSHL5.1*), co-localizing at the *G* locus (peach/nectarine morphological marker, *p*-value 1.17e^−07^) (Fig. 7). Putative pleiotropic effects of *G* trait on *SH*_*L*_ were also evident after accounting for *qSHL*/*Fs6.1* haplotypes, although only in H1H2 and H2 background (Fig. 7). Naive GLM model was also rerun for *Fs* trait, also identifying a signal at *G* locus but with a lower *p*-value (1.29e^−05^). However, when accounting for *qSHL*/*Fs6.1* haplotypes, the effect of *G* trait on *Fs* was only significant in the H2 background (Fig. 7).

Regarding the major QTL for *Fs* found on chr 4, low LD levels were found around the top associated signal at Peach_AO_0424020. Interestingly, this SNP has a low MAF (0.09), with the minor allele homozygous or heterozygous in all ultra-early maturing accessions (i.e., 10 accessions with a MD range from 142 to 162 Julian days). This marker seems associated with a decrease of *Fs*, although differences were only significant for homozygous genotypes (Supplementary Fig. 8). Indeed, at the whole panel level, no significant relationship was found between maturity date and size.

### Mapping of a major QTL for fruit size and shape from ornamental breeding progenies

**Fig. 8:**
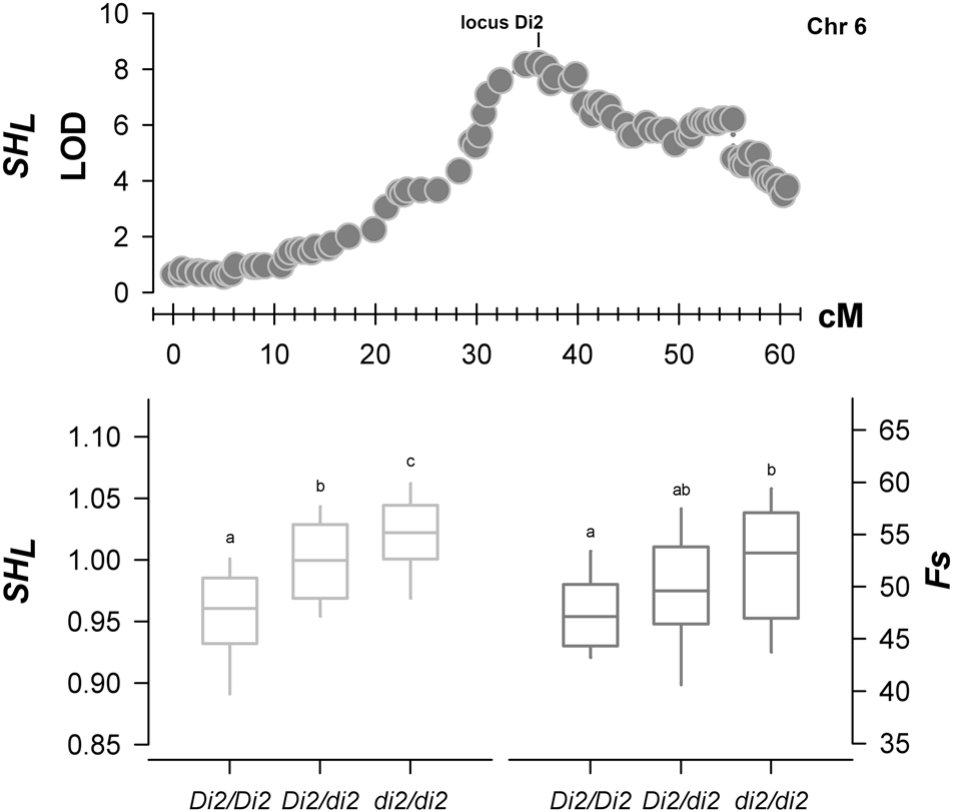
Linkage mapping for fruit longitudinal shape (*SH*_*L*_) in WxBy progeny. Significance of QTL analysis was calculated using LOD score.Map positions on chromosome 6 is also shown. Box-plot of markers-trait association between the *Di2* marker, fruit longitudinal shape (*SH*_*L*_) and size (*Fs*) in WxBy progeny using averaged yearly data. Letters indicate significant differences between segregating classes (*p* < 0.01) according to one-way ANOVA

*SH*_*L*_ and *SH*_*T*_ traits were also phenotyped in an F_2_ progeny (WxBy) from the cross of ornamental peach ‘NJ Weeping’ [W, round-ovate (*SH*_*L*_ = 0.95), extra-small size (*Fs* = 42.9 mm)] × ‘Bounty’ [By, slightly ovate (*SH*_*L*_ = 1.00), medium-large size (*Fs* = 67.2 mm)]. In WxBy, *SH*_*L*_ and *SH*_*T*_ both showed a normal but slightly negatively skewed distribution. Correlation between the two seasons was high for *SH*_*L*_ and low for *SH*_*T*_ (*r*-squared of 0.67 and 0.34, respectively). Linkage analyses in WxBy map showed a major QTL signal for *SH*_*L*_ in the middle of chr 6, peaking at the *Di2* morphological marker (24,074,355 bp) with a maximum LOD of 8.3% and 27.8% of explained variance (Fig. 8). This QTL (named *qSHL6.2*) was stable when considering yearly data and co-localized within the dominant *Di2* locus that controls flower petal number. Indeed, segregation of the *Di2/di2* locus was significantly associated with *SH*_*L*_ values, which additively increase from ovate in homozygous double-flower (*Di2/Di2*) individuals (*SH*_*L*_ around 0.95) to progressively rounder shapes in heterozygous (*Di2/di2*) double-flower and single flower ones (Fig. 8). In contrast, effects of *Di2* locus on fruit size were most evident when comparing single versus homozygous double-flower (*Di2/Di2*).

## Discussion

### Phenotypic variability of fruit size and shape traits in a peach germplasm collection

The main peach shape descriptors reported in the UPOV guidelines are based on the qualitative ranking of fruit into discrete classes through visual analyses. Such subjective scoring systems pose limitations for genetic analyses. Therefore, a first goal of this work was the collection of quantitative phenotypic data based on objective, standard indices for shape traits, determinable through measurements with a digital caliper and/or image-based software. Other than allowing precise quantification of phenotypic variation, such indices have the advantage of being relatively easy to collect and comparable among different studies. Among other approaches to evaluate shape traits, 2D or 3D imaging-based approaches involving elliptic Fourier descriptors (EFD) have been also adopted in fruit species^40–43^. In peach, EFD has been mainly used to also define the influence of the position of the center of gravity, curvature, and degree of roundness^44^. However, width-to-height and length-to-width ratios seem to account for most of the variability for fruit shape in peach^5,45^, as also observed in our collection.

In several species, fruit shape is often altered following the size alteration and viceversa^46^. Among the various non-genetic factors affecting size, fruit load is probably the most important, as it can account up to 84% of size variability within a single accession^47^. As an example, fruit size in a typical large-sized peach commercial cultivar (e.g., ‘July Prince’) can range from 50 until 90 mm as a sole function of the fruit load^47^. This range is close to the whole variability measured in our collection (about 50–80 mm). Imposing two divergent fruit load regimes in a subset of accessions clearly indicated that thinning-induced increase of fruit size also tended to modify shape, particularly, increasing *SH*_*L*_. Thus, in order to minimize confounding effects due to source-limitations, we set a light fruit load (0.7–1 fruits per cm^2^ of TCSA), ideally allowing accessions to express their maximum fruit growth potential. The tight control of fruit load allowed the reduction of year-to-year differences within accession, resulting in 0.69 *Fs* correlation among the two years of assessment. Clearly, specific recommendations for thinning intensity in terms of fruit number per TCSA (such as the range of fruit load adopted in this work) are not universally valid for all environments and cultivation conditions; nevertheless, management and specific fruit load conditions are crucial for an accurate assessment of peach fruit size and shape, as well as to ensure a reliable comparison across studies. Also, estimating genotype × management (i.e., fruit load) interactions at the whole panel level (e.g., by setting different crop load levels) may be a matter of future studies, although imposing different fruit loads is not always feasible in breeding progenies or in collections of genotypes characterized by different growth vigor and productive habitus.

For fruit size (*Fs*) trait, in other studies at peach germplasm-level^21,34^, the range of phenotypic variability has not been reported in detail, hindering a direct comparison with our work. In 14 non-flat intra-specific peach breeding progenies, average population *Fs* values ranged from about 65 to 80 mm^5^, as also observed in our work, while in a BC_2_ peach × *P. davidiana* progeny and BC_1_ ‘Texas’ × ‘Earlygold’ the lower limit of the range reached less than 40 mm^22,48^.

Regarding shape, among the measured indices, *longitudinal shape* (*SH*_*L*_) (i.e., width-to-height ratio) and *transversal shape* (*SH*_*T*_) (i.e., width-to-length ratio) are particularly relevant in peach breeding, since they subtend to ovate, round or oblate forms of the fruit and its regularity, respectively^2^. A germplasm-wide analysis of these traits in peach has not been reported in the literature. In our work, the phenotypic variability recorded for these two shape traits can be considered as representative of the intra-species variability in Occidental accessions. Although additional diversity may be present in Oriental materials, the observed ranges of variation for *SH*_*L*_ and *SH*_*T*_ (0.86–1.19 and 0.96–1.13, respectively) are comparable to those reported in a pool of accessions including also peach hybrids with wild species, such as davidiana (0.95–1.08 and 0.96–1.07)^45^ or almond^48^. Most accessions in our panel (particularly those recently released) showed values for *SH*_*L*_ attributable to a nearly round form or slightly oblate, as a consequence of the strong selection activities towards this consumers’ preferred attribute. While *SH*_*T*_ showed a more limited range of variability, this parameter was less stable within the two years of evaluation, implying more pronounced effects of environmental conditions compared to *SH*_*L*_. *SH*_*T*_ is visually associated to fruit shape regularity and tends to be strongly altered by morphological defects such as triangular-shaped fruits and/or prominent suture (both frequently associated to the occurrence of internal endocarp splitting)^49^. Protruding tips and sutures can be bruised during handling and shipping, and are thus undesirable traits for commercial peaches^50^. The presence of genotypes more prone to these defects has been variously reported, as confirmed in our panel by the out-ranking accessions (such as ‘Pillar’ or ‘Groc d’alto’) with a high incidence of triangular-shaped fruits. These accessions may be used as cross-parents for studying the trait in a more restricted genetic background, although broad-sense heritability of suture prominence seems to be very low^51^. Longitudinal Shape Triangularity index (*ST*_*L*_) was developed to quantify the degree of fruits ellipticity. The heart shape is considered a fruit defect, particularly when highly accentuated (*ST*_*L*_ above 1.20) and associated with a pronounced tip. Most of the accessions showed values around 1.10 for this index and a low frequency of extreme phenotypes. The degree of ellipticity and tip prominence tend to increase in sub-tropical peach cultivations, mainly as a consequence of insufficient chilling accumulation but also elevated post-bloom temperatures^18,52,53^. Not surprisingly, this shape trait has considerable importance in low-chill cultivar development^17^, although the selection is hindered by year-to-year variability (as also observed in our panel) and strong genotype-by-environment interactions^19^. Growing condition of our collection, ensuring complete satisfaction of cold requirement even in the most demanding accessions, are probably the reason for the poor variability observed for tip morphology or protruding tip incidence. An interesting accession (‘Pesca a Cuore Capuozzi’) with heart shape and the evident tip could be useful to analyze these traits in temperate climates. The limited range of variability and low yearly stability found for other shape traits, such as proximal indentation (*Δ*_*CH*_) width and height of stalk cavity (α, *H*_*ST*_) or indentation angle (β), will require additional studies in other germplasm collections for clarifying the actual presence of relevant phenotypic diversity in peach.

### Genetic basis of non-flat shape variability in peach

To our knowledge, this work is the first effort to characterize the genetic architecture of fruit shape variability in non-flat peach cultivars. Association mapping revealed several genomic loci associated with fruit shape; information about their positions will be useful as a starting point for extending research to other germplasm collections. Cumulative heritability explained by GWAS loci was moderately high for longitudinal shape (*SH*_*L*_) and fruit size (*Fs*) (around 0.60) and low for the other shape traits. A major effect locus for longitudinal shape (*SH*_*L*_) was identified at the beginning of chr 6 (*qSHL/Fs6.1*) within an LD region of about 1.2 Mb. Allelic variation at this locus was largely represented by two haplotype blocks (named H1 and H2) accounting for over 30% of the phenotypic variance for *SH*_*L*_ in the collection panel, with H1, H1H2, and H2 genotypes, respectively, associated to ovate, round and oblate shapes. Interestingly, homozygosity for the ovate-associated H1 haplotype was underrepresented compared to the other two genotypes (only 8%), suggesting at least in our collection, a negative selection toward this allelic configuration. The effects of this QTL were further validated in two progenies (BxO and CxEL^2^), where segregation of *qSHL/Fs*6.1 explained most of the observed phenotypic variability for *SH*_*L*_.

A second major QTL for *SH*_*L*_ (*qSHL5.1*) was found co-localizing at locus G. The correlation among traits due to pleiotropy or strong linkage among QTLs seems to be an important driver of fruit quality multi-trait evolution. For example, a strong linkage between flat trait and a reduction of fruit weight has been reported in peach progenies^5^, also supported by co-localization of locus *S* with fruit mass QTLs^10,54^. As observed in bud sports, peach/nectarine mutations would appear to have a pleiotropic or strong linkage effect on some traits, particularly fruit size and shape^55^. The effect of this locus was clear when comparing longitudinal shape in nectarine versus peach accessions, the first showing significantly lower *SH*_*L*_ value, and more round shape. Putative pleiotropy of the nectarine trait seems also evident when accounting for *qSHL/Fs*6.1 segregation, although not significant in the H1-ovate background. The exclusion of nectarines in a multi-progeny study significantly reduces heritability for fruit size, from 0.40 to 0.09^19^. However, G trait effect on *Fs* was elusive in our panel. Given its relevance for breeding, the role of nectarine trait on both *SH*_*L*_ and *Fs* should be more specifically addressed, particularly in progenies segregating for both *G* and *qSHL*/*Fs6.1* loci. Apart from these two major loci, other QTLs were also identified by GWAS on chr 1 and 4, although the first only by two of the three tested algorithms. However, the low LD level found around top associated SNPs did not allow haplotypes inference and reliable estimates of the contribution to *SH*_*L*_ variability.

The dissection of fruit size has been reported in several studies. In a pedigree-based analysis, QTLs on chr 6 (in the interval 3.7–6.3 Mb) and 7 (6.6–9.5 Mb) were stable across seasons, and a QTL on chr 5 (2.5–3.2 Mb) was detected in one year^21^. In another study involving materials from the UC Davis Breeding Program including introgressions from wild relatives (*P. argentea*, *P. mira*, *P. davidiana*, and almond), QTLs for *Fs* were identified on chr 2, 3, and 7 although in a different position compared to the previous analysis (21–22.5 Mb)^34^. In WxBy progeny, a QTL on chr 7 explained the majority of size variation, but it was located in a different interval compared to the previous studies (maximum LOD for SNP_IGA_776826 at 15.2 Mb), with additional stable loci detected on chr 1 (11.8 Mb) and 3 (13.7 Mb)^33^. In the BxO progeny, a major QTL for *Fs* perfectly co-localizing at the *qSHL/Fs6.1* locus was detected in the same region previously reported on chr 6^34^. The range of fruit size segregation (from less than 50 to more than 70 mm) and the high proportion of explained variance (about 60%) further support the relevance of the QTL in this genetic background. The co-localization of *Fs* and *SH*_*L*_ QTLs in the BxO progeny suggests a pleiotropic effect of *qSHL*/*Fs6.1*, however, recombination events around this locus did not allow to confidently narrowing the interval (data not shown). Therefore, whether *qSHL*/*Fs6.1* harbors multiple QTLs or a single QTL with pleiotropic effects remains an important question for future research. Association of segregating haplotypes at *qSHL/Fs6.1* and *Fs* was less evident in the CxEL^2^ progeny and the germplasm panel, suggesting a more complex architecture of this trait compared to *SH*_*L*_. Indeed, other loci were detected by GWAS, with the most significant signal at the proximal end of chr 4. This QTL seems to be mainly linked to the small fruit size characterizing early maturing accessions (such as ‘Lucrezia’, ‘Borgia’, ‘Elios’) suggesting either the presence of a linkage between *Fs* and maturity date (MD) loci or pleiotropic effects of MD. MD effects on fruit size or mass have been already reported in peach and other species, supported by an overall moderate correlation in several breeding progenies^8,37,56^. However, excluding very early maturing accessions, the relationship between *Fs* and *MD* in our panel was not significant. Moreover, the position of this QTL does not coincide with a major MD locus previously reported^57^.

The particular genetic background of the WxBy progeny (derived from ‘NJ Weeping’ PI91459, an ornamental tree selected based on flower rather than fruit phenotype) allows to expand the diversity associated with fruit size and shape and evaluate the re-introgression of wild-related traits in a modern breeding background (i.e., ‘Bounty’). A previous linkage analysis of *Fs* variation in WxBy has uncovered a complex genetic inheritance, controlled by several major and minor QTLs^33^. Using the same dataset, a major QTL (*qSHL6.2*) for *SH*_*L*_ was uncovered at the *Di2* locus, controlling the double-flower trait. This locus seems to be regulated by a deletion of the miR172 target site in the AP2/TOE-type family gene *PETALOSA* altering flower morphology through the development of supernumerary petals^58^; additional studies should be performed to explore the possible pleiotropic effects of this variant, as previously demonstrated for heat-requirements in the same background^59^. Indeed, this aspect would provide insight into the link between environmental growing conditions (i.e., phenology) and fruit morphology. In apple, the AP2-miR172 module influences fruit size, since a transposon insertion in a miRNA172 allele co-located with a major QTL for fruit size in large-sized cultivated and wild progenitor apple species, whereas over-expression of miRNA172 in transgenic apple significantly reduces fruit size^60^.

For other shape traits (*α*, Δ_CH_, *ST*_*L*_ or *SH*_*T*_), our genomic scan did not allow to find stable and highly significant loci, as they seem to be affected by the environment (and, probably, genotype by environment and/or by management interactions). Unraveling the genetic bases of such traits will require additional studies and particular experimental designs (such as replicated multi-site collections)^61^. Also, novel phenotyping methods should be tested, including the use of machine learning or 3D imaging in morphometric approaches, as tested in other fruit species^43^. Considering the high within-tree variability of these traits, quantification in terms of incidence (rather than average value) could be useful to improve phenotyping resolution, although this will require the analysis of a large number of fruits, which is not always feasible in large collections.

### Putative candidate gene associated with fruit size and shape on chromosome 6

A relevant number of QTLs affecting fruit size and shape have been cloned in plant species, revealing the conserved function of several gene classes on the control of these traits in agriculturally important species^1^. In tomato, a model species for fleshy fruits, the diversity among cultivars is largely explained by at least six QTLs: *fasciated* encoding a YABBY family member controlling locule number leading to flat shape^62^; *sun* encoding a member of the IQ domain family of calmodulin-binding proteins leading to fruit elongation^63^; *ovate* encoding a member of the Ovate Family Proteins (OFP) involved in transcriptional repression leading to fruit elongation^64^; *lc* putatively encoding the ortholog to WUSCHEL controlling meristem size and locule number^65^; *fw2.2* encoding a member of the Cell Number Regulator family^66^ and *fw3.2* encoding a cytochrome P450 enzyme of the CYP78A class ortholog to KLUH^67^. Other QTLs have been recently cloned, such as ENO, an AP2/ERF transcription factor that regulates tomato fruit size by affecting floral meristem development^68^ or *sov1*, encoding another member of the OFP family (*SlOFP20*)^69^. Apart from their functional conservation, the presence and effect of genetic variation associated with these genes remained to be elucidated in peach and other stone fruits. Members of the previously described gene families localize within confidence intervals of major QTLs in other taxa, suggesting that organ shape and size variability may have a common genetic basis^70^. For example, a candidate gene of the FW2.2/CNR clade, *PavCNR12* co-localize with a major QTL on chr 2 explaining the highest portion of the phenotypic variation in modern cherry germplasm^71^. In peach, a 1.7 Mb chromosomal inversion putatively affecting an OFP gene (*PpOFP1*) has recently been identified as the most promising candidate for flat trait^39^. The whole‐genome sequence of peach^72^ is a useful resource for the identification of candidate genes underlining QTLs for fruit size and shape. Focusing on chr 6 QTLs, other than the *PpOFP1* gene, two additional members of the OFP gene family, *PpOFP5* and *PpOFP15*, encoded by transcripts *Prupe.6G042700.1* (at 3.13 Mb) and *Prupe.6G042800.1* (3.14 Mb)^73^ co-localize at *qSHL/Fs6.1*, representing promising candidates for this major locus (Supplementary Fig. 9). Further exploration of allelic variants at this locus and/or candidate genes represent an interesting development of this research. In addition to the *PETALOSA* gene^59^, also a FW2.2/CNR clade member (*PpCNR20*) encoded by transcript *Prupe.6G240600* (at 23.9 Mb) was present within the 2-LOD interval of *qSHL6.2* (Supplementary Fig. 9). Clearly, further work is needed to validate the presence of genetic variability at these gene loci, and their possible involvement in controlling these traits.

## Conclusions

This work provides a dissection of fruit size and shape diversity in a peach collection. Collectively, the range of variability reported for shape attributes provides a reference for their comparison in other germplasm collections. This work also highlighted the importance of fine-tuning fruit load management for the accurate assessment of size and shape traits. The identification of major loci controlling fruit shape and size, and associated markers, provides useful information for breeding and opens opportunities for more in-depth studies aimed at improving their resolution and identifying underlying variant(s).

## Materials and methods

### Plant materials

The panel used in this study is comprised by 172 ‘round’ and 11 ‘flat’ accessions derived from MAS.PES peach germplasm collection located in the experimental farm ‘M. Neri’ of CRPV (Centro Ricerche Produzioni Vegetali) in Imola (Emilia-Romagna region, Italy) (Supplementary File 2). Trees were grafted onto ‘GF677’ rootstock, trained according to open vase system, and regularly spaced at a distance of 4 × 2.5 (within and between rows, respectively). Trees were managed according to standard cultural practices for irrigation, fertilization and pruning. Fruits were thinned within 40–60 days after bloom, setting a crop load proportional to tree vigor, estimated by Trunk Sectional Cross-Area (TCSA). Crop load (number of fruits per cm^2^ TCSA) was set based on a previous work^74^ and taking into account average yield per tree density in close commercial orchards. The F_1_ cross (BxO) from ‘Bolero’ × ‘Oro A’ parents comprising 126 seedlings located in the experimental field of the University of Milan, Azienda Didattico Sperimentale ‘F. Dotti’ (Lodi, Italy)^75^. The F_2_ cross (WxBy) from ‘NJ Weeping’ × ‘Bounty’ parents comprising 123 seedlings located at the CRPV orchards in Castel S. Pietro (Bologna, Emilia-Romagna region)^33^. The F_2_ cross CxEL^2^ derived from self of F_1_ CxEL individual #071 comprised 100 individuals located at the CRPV orchards in Imola (Bologna, Emilia-Romagna region). BxO and CxEL^2^ progenies were grafted onto GF677 rootstock, while WxBy were own-rooted, and all planted at distance of 1 × 4 (within and between rows, respectively) and trained as slender spindle (one stem with short lateral scaffolds). In the three progenies, fruit thinning was performed as previously described^33^, leaving 1–3 fruits per fruiting shoot and harvesting the 10 largest fruits from each tree. Climatic conditions of growing areas allow the complete fulfillment of chilling requirement also in high-chill accessions.

### Phenotyping

Fruits were collected at commercial maturity on seasons 2018–2019 for the accession panel, on 2011–2012 for WxBy, 2013–2014 for BxO and 2017 for CxEL^2^ progenies. Morphological traits were assessed in at least 10 fruits from each tree adopting previous developed indices^76^. Maximum fruit height (*h*), width (*w*), and depth (*d*) were determined by a digital caliper, and the results given in millimeters. The degree of suture indentation in the transversal section (*γ*) was visually scored. The indices *shape triangle index (STi*), *width of stalk cavity* (*α*); *height of stalk cavity* (*H*_*ST*_), *proximal indentation* (*Δ*_*CH*_), and *indentation angle* (*β*), were evaluated through image analysis: ten fruits were transversally and longitudinally sectioned, placed on a rigid graph paper in the center of the camera’s field of view, capturing two RGB color images for accession. Images were manually processed using ImageJ software^77^, after calibration performed using the set scale function. Descriptive statistic of data was performed in R environment. Correlation between traits was analyzed using the Spearman correlation coefficient. Normal distribution was tested using the Shapiro–Wilk test (*p* < 0.05).

### Genotyping and population structure

The novel released 18K SNP array peach, composed by the IPSC peach 9K SNP array^78^ plus a 9K SNPs add-on derived from high-coverage whole-genome re-sequencing data^79^ was used to genotype the analyzed panel of 172 accessions and CxEL^2^ progeny. Genotyping data were filtered for marker missing rate <10% and minor allele frequency (MAF) > 5%, finally retaining a total of 12,473 high-quality SNPs for GWA analysis. The Peach Genome assembly V2.0 was used as a reference for SNP marker positions^69^. BxO and WxBy progenies were genotyped using the IPSC peach 9K SNP array, as previously described^33,57^. Population substructure was inferred using the model-based clustering algorithm ADMIXTURE v1.22, Successive values of K (a priori genetic clusters) were imposed, choosing the K which maximize the predictive accuracy through a cross-validation procedure. Principal Component Analysis (PCA) was also performed using the full set of filtered SNPs through the R function *prcomp*. The optimal number of PCs to be included for the considered phenotype was determined by using Bayesian information criterion (BIC). Genotyping and map construction in BxO and WxBy progenies was previously described^33,57^.

### Genome-wide association and population structure analyses

For association analysis, different algorithms were adjusted using a *Q* = 2 matrix as covariate: Mixed Fixed and random model Circulating Probability Unification (FarmCPU), Multi-locus Mixed Linear Model (MLMM), and linkage disequilibrium iteratively nested keyway (Blink). A naive Generalized Linear Model (GLM) without correction for population structure was also used. The performance of all tested GWA algorithms was evaluated by comparing the observed vs. expected *p*-values under null hypothesis through quantile–quantile (QQ) plot inspection. For association significance, thresholds were calculated based on Bonferroni correction for a type I error rate of 0.05 (4.01e^−06^) and less stringent (trait-specific) permutation tests. SNP-based broad-sense heritability was estimated by GREML method using GCTA tool after fitting GWAS top SNPs. Intra-chromosomal LD patterns were calculated using r-squared correlation for all pairwise SNPs comparisons and visualized using HAPLOVIEW v4.2. SNP haplotype blocks at *qSHL/Fs6.1* were deduced through identical-by-state (IBS) phasing.

### QTL mapping and haplotype-trait association

Linkage maps and genotypic data used for QTL analysis in BxO and WxBy progenies were already available from previous studies. QTL analyses were carried out using the MapQTL 6.0 software^80^. According to normality distribution (Shapiro–Wilk test) the non-parametric Kruskal–Wallis (KW) rank-sum test or interval mapping (IM) were used to search for phenotype-marker associations. A QTL was considered significant for LOD values higher than 3 in IM and *p* = 0.005 for the individual test in KW. In case of not-normal data, IM approach was used for an approximate estimate of the QTL-explained percentage of phenotypic variance. Association of *qSHL/Fs6.1* haplotype class and *SH*_*L*_ and *Fs* in the BxO and CxEL^2^ progenies was determined using in non-recombinant individuals. Comparison among phenotypic values of each segregating class was estimated by ANOVA, Kruskal–Wallis, and two-tailed Student’s test (*p* < 0.01). Statistical analyses were carried out in an R environment.

## Supplementary information

Supplemental File 1

Supplemental File 2

Supplementary materials description

Supplemental Figure 1

Supplemental Figure 2

Supplemental Figure 3

Supplemental Figure 4

Supplemental Figure 5

Supplemental Figure 6

Supplemental Figure 7

Supplemental Figure 8

Supplemental Figure 9

## Data Availability

Raw data and R script for statistical analysis are available at link https://github.com/marcoc83/Rnotebook.git. Additional data are available upon request.
