# Supplementary material for: Genetic and phenotypic analyses reveal major quantitative loci associated to fruit size and shape traits in a non-flat peach collection (P. persica L. Batsch)
Source: Hortic Res. 2021 Nov 1;8:232. doi: 10.1038/s41438-021-00661-5 (PMC8558339; doi:10.1038/s41438-021-00661-5)
Supplement: Supplementary file 3 — Supplementary materials description [file 41438_2021_661_MOESM3_ESM.docx]

**Supplementary materials description**

**Supplemental Figure 1**. Pearson’s correlation among yearly data for all analyzed size and shape traits.

**Supplemental Figure 2**. Frequency distribution of two-years averaged data for *longitudinal* *shape triangle index* (*ST_L_*), cheeks asymmetry (*Δ_CH_*), *width of stalk cavity* (α) and *height of stalk cavity* (*H_ST_*) in the collection panel.

**Supplemental Figure 3**. A) Cross-validation for each a priori cluster number (K values) as inferred in Admixture. B) Structure plot of the analyzed panel for the optimal number of a priori genetic clusters (K = 2), with ancestry proportion on the Y-axis.

**Supplemental Figure 4**. Principal component analysis (PCA) of morphological traits and genetic structure of the collection panel. The first two components (Dim1 and Dim2) are shown, accounting for 31.4 and 20.4% of explained variance, respectively. Accessions were grouped according to the population structure.

**Supplemental Figure 5**. Manhattan and QQ-plots of the -log10 *p*-values estimated for the monogenic fruit flat shape trait (locus S) using FarmCPU algorithm. Horizontal line indicates the Bonferroni-adjusted threshold (4.01e^-06^).

**Supplemental Figure 6**. Pattern of linkage disequilibrium around SNP_IGA_609531 and SNP_IGA_605104, respectively associated to fruit *longitudinal shape* (*SH_L_*) and *size* (*Fs*).

**Supplemental Figure 7**. Linkage mapping for fruit *longitudinal shape* (*SH_L_*) and *size* (*Fs*) traits in BxO progeny. According to trait distribution in each progeny, significance was calculated using LOD and K-score respectively for *SH_L_* and *Fs*. Map positions on chromosome 6 and two-year averaged phenotype distributions are also shown.

**Supplemental Figure 8**. Boxplots of the association between Peach_AO_0424020 genotypes (AA, AG and GG) on chromosome 4 with fruit *size* (*Fs*) in the collection panel. Letters indicate significant differences between segregating classes (*p* < 0.01) as inferred by ANOVA on ranks. Orange bar indicates maturity date (MD) range.

**Supplemental Figure 9.** Most relevant candidate genes for fruit size and shape identified on chromosome 6, and co-localizing within the confidence intervals of q*SHL*/*Fs6.1* and q*SHL6.2* QTLs*.*

**Supplemental Files**

**Supplemental File 1**. LD blocks at q*SHL*/*FS6.1* locus in the accessions panel and cross-parents of BxO, CxEL^2^ and WxBy progenies.

**Supplemental File 2**. List of peach accessions and selection used in this study and phenotypic data.
