# Supplementary figures and images for: Genetic and phenotypic analyses reveal major quantitative loci associated to fruit size and shape traits in a non-flat peach collection (P. persica L. Batsch)
Source: Hortic Res. 2021 Nov 1;8:232. doi: 10.1038/s41438-021-00661-5 (PMC8558339; doi:10.1038/s41438-021-00661-5)

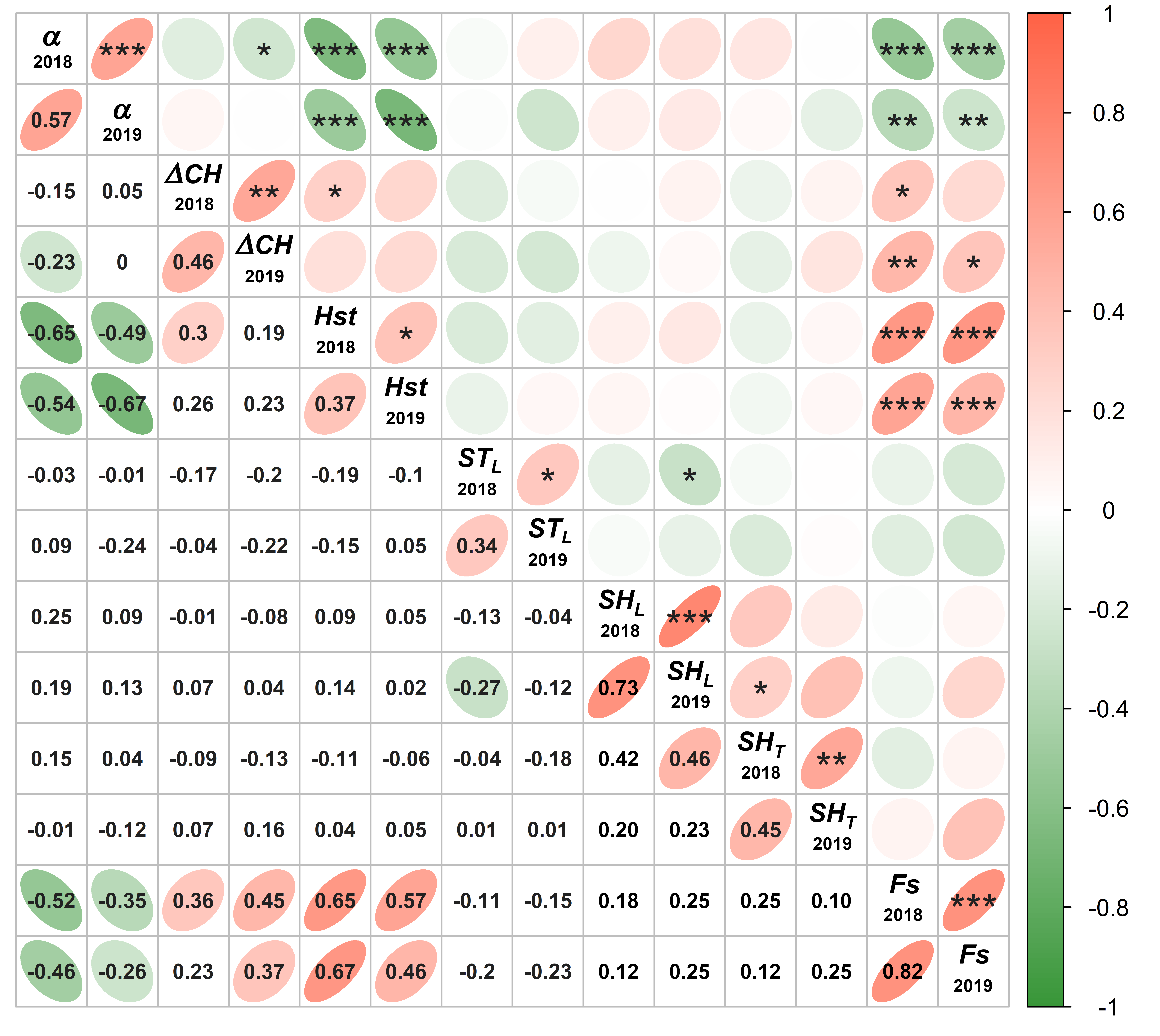

Supplement: Supplementary file 4 — Supplemental Figure 1 [file 41438_2021_661_MOESM4_ESM.png]

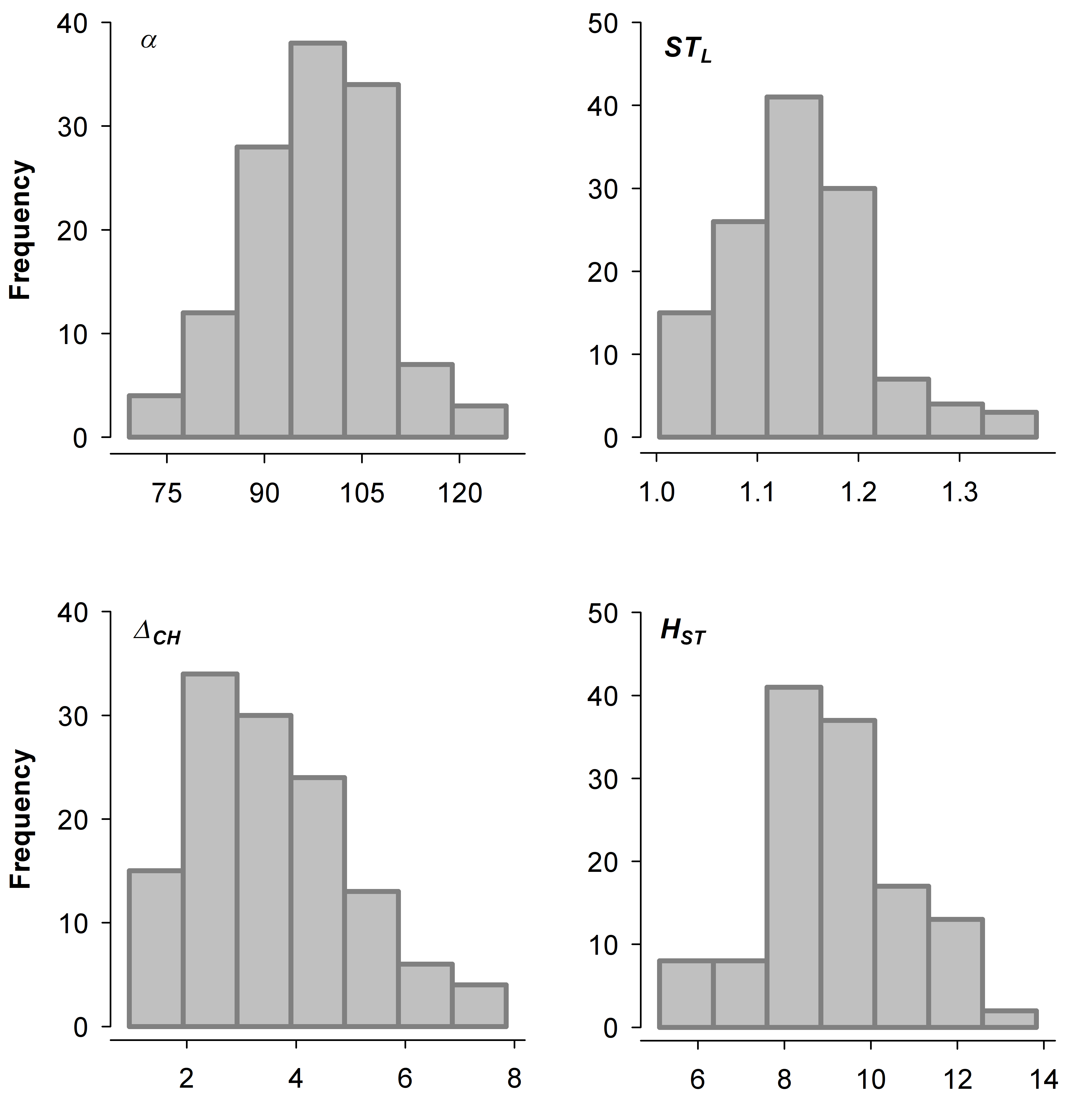

Supplement: Supplementary file 5 — Supplemental Figure 2 [file 41438_2021_661_MOESM5_ESM.png]

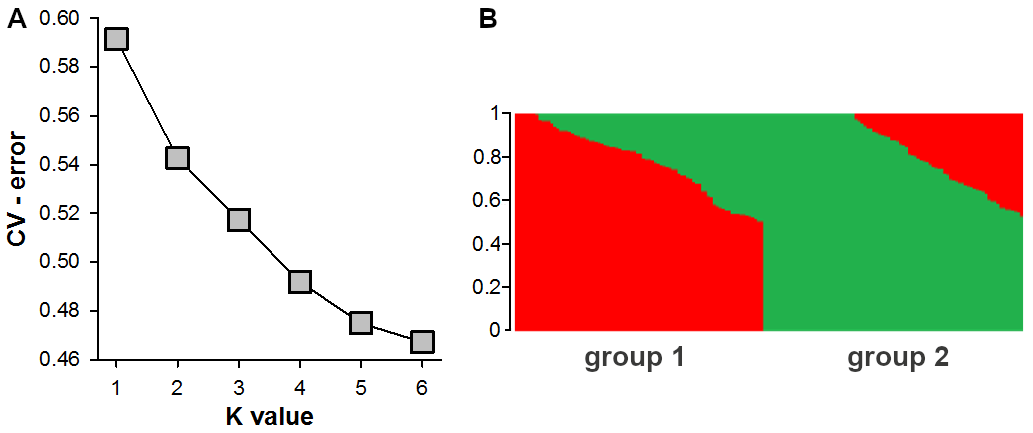

Supplement: Supplementary file 6 — Supplemental Figure 3 [file 41438_2021_661_MOESM6_ESM.png]

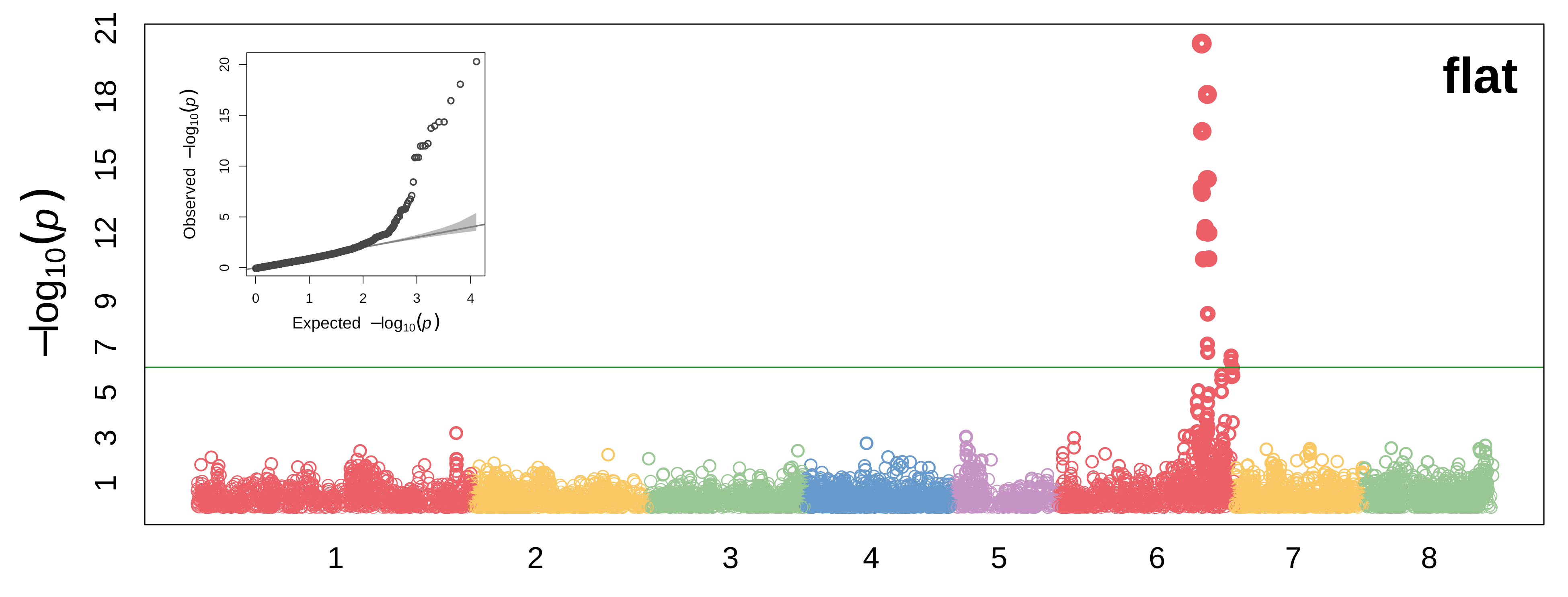

Supplement: Supplementary file 8 — Supplemental Figure 5 [file 41438_2021_661_MOESM8_ESM.png]

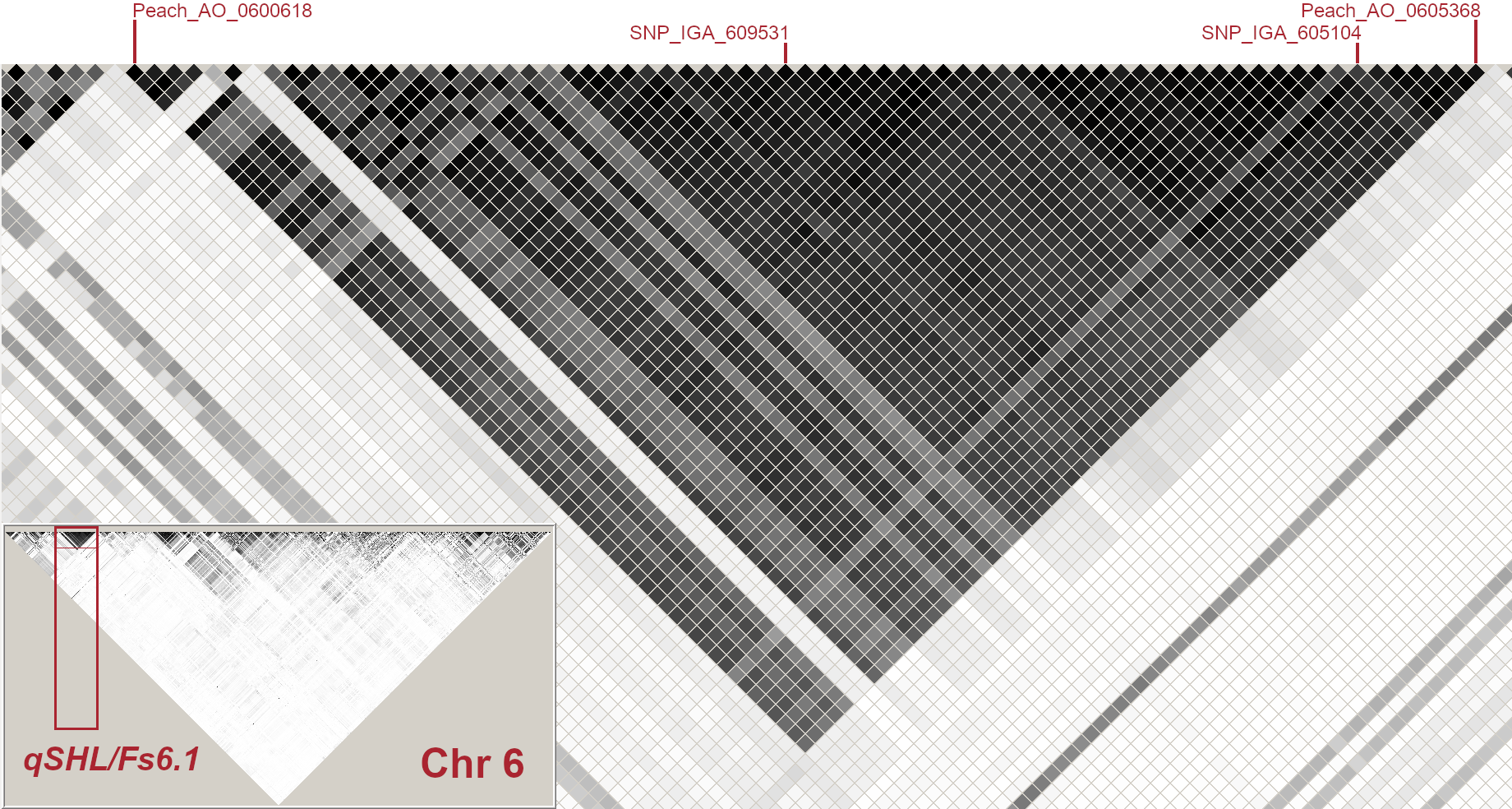

Supplement: Supplementary file 9 — Supplemental Figure 6 [file 41438_2021_661_MOESM9_ESM.png]

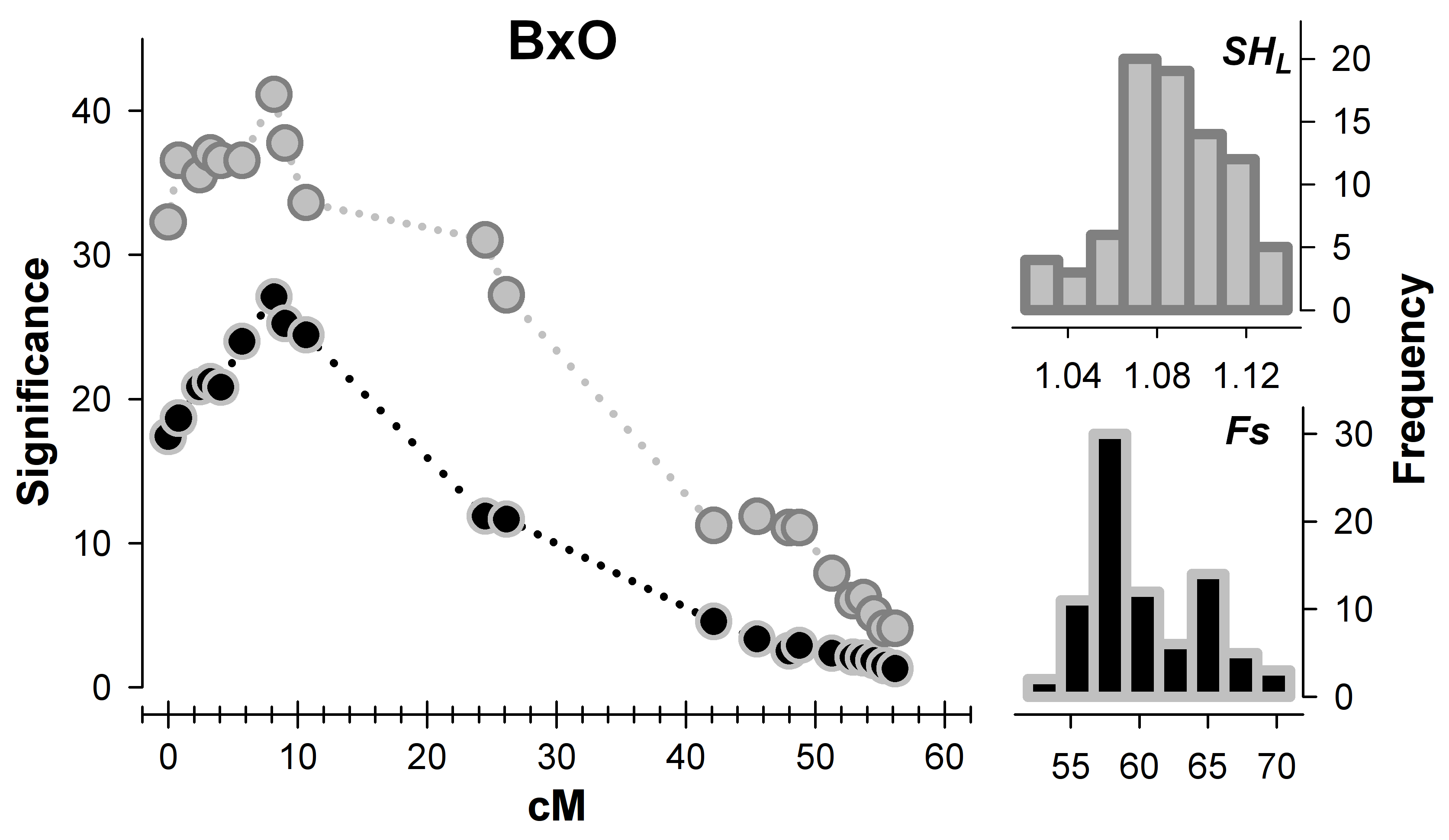

Supplement: Supplementary file 10 — Supplemental Figure 7 [file 41438_2021_661_MOESM10_ESM.png]

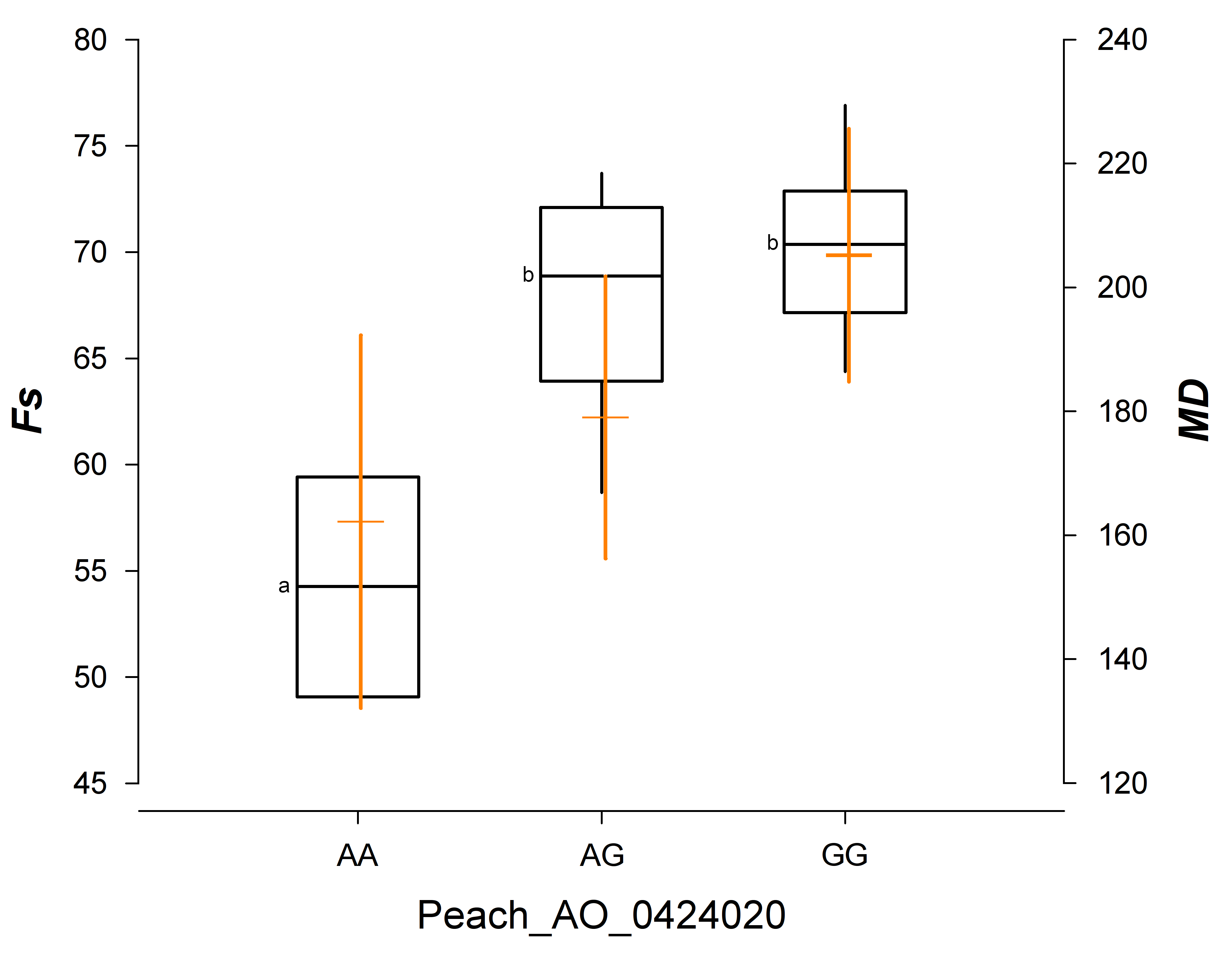

Supplement: Supplementary file 11 — Supplemental Figure 8 [file 41438_2021_661_MOESM11_ESM.png]

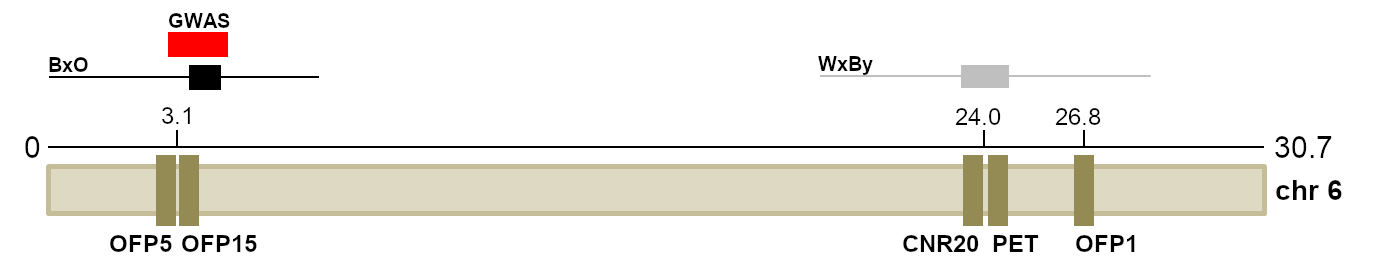

Supplement: Supplementary file 12 — Supplemental Figure 9 [file 41438_2021_661_MOESM12_ESM.png]
